# A Novel Flavonoid C‑Glycosides Integrated Tablet for Improved Dissolution, Pancreatic Repair, and Insulin Mediated Glucose Regulation in Type 2 Diabetic Rats

**DOI:** 10.1021/acsomega.5c06592

**Published:** 2025-09-17

**Authors:** Abdul Rahim Muhammed Jasim, Anithakumari Aswathy Krishna, Beena Levakumar Abhirami, Alaganandam Kumaran, Chun-Hui Chiu

**Affiliations:** † Agro and Food Processing Technology Division, CSIR−National Institute for Interdisciplinary Science and Technology (NIIST), Thiruvananthapuram 695019, Kerala, India; ‡ Academy of Scientific and Innovative Research (AcSIR), Ghaziabad 201002, India; § Research Center for Food and Cosmetic Safety, College of Human Ecology, 63113 Chang Gung University of Science and Technology, Taoyuan City, 333324, Taiwan; ∥ Department of Nephrology, Chang Gung Memorial Hospital, Taoyuan City 333008, Taiwan

## Abstract

Flavonoid C-glycosides (FCGs) are polyphenols with a stable C–C glycosidic bond, known for antidiabetic and antioxidant effects. However, their poor water solubility and low dissolution limit oral bioavailability. To overcome these limitations, a tablet formulation (*Cassia mimosoide*-formulation batch 4, CM F4) was developed using a FCG-rich ethyl acetate extract of *Cassia mimosoides* L. (CM-EA), a plant traditionally known for its antidiabetic properties. The optimized formulation improved tablet disintegration, stability, and significantly enhanced FCG release, confirmed by *in vitro* dissolution and HPLC analysis. In high-fat diet and streptozotocin-induced diabetic rats, CM F4 reduced fasting blood glucose, improved glucose tolerance, restored pancreatic β-cell architecture, and increased insulin levels. It also modulated key carbohydrate-metabolizing enzymes and upregulated insulin signaling proteins, particularly PI3K/AKT and AMPK pathways. This study presents CM F4 as a novel tablet formulation that enhances the dissolution and *in vivo* antidiabetic efficacy of plant-derived FCGs, offering a promising phytopharmaceutical approach for type 2 diabetes management.

## Graphical abstract

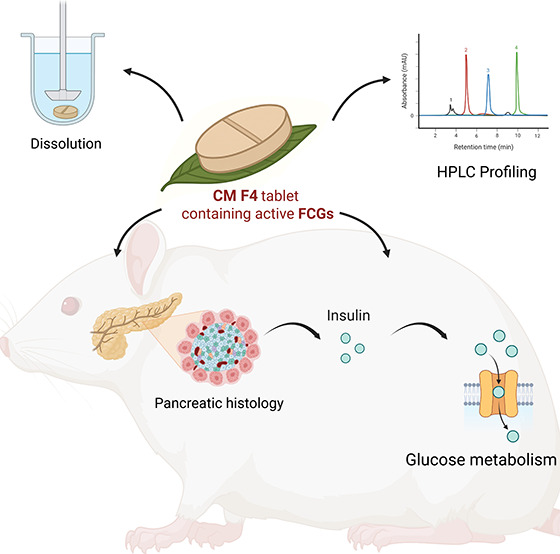

## 1 Introduction

Type 2 diabetes mellitus (T2DM) is a widely seen manifestation of diabetes that occurs majorly due to alterations in lifestyle and insufficient physical activity, resulting in the malfunction of beta cells and the development of insulin resistance.^1^ The prevalence of diabetes has increased, making it a major health problem that affects a significant portion of the global population. In addition to the failure of β cells, the primary pathophysiological mechanism that propels the progression of T2DM is the resistance of target tissues to insulin, which is frequently accompanied by abnormal insulin secretion.^2^ One of the main factors that play a significant role in the emergence of insulin resistance is obesity, a condition that is primarily caused by the adoption of a high-fat diet (HFD) that aligns with Western dietary patterns. This is further complicated by a sedentary lifestyle with minimal physical activity.^3^

T2DM continues to pose a significant global health burden, with estimates projecting its steady rise in prevalence due to increasing urbanization and lifestyle changes. While looking to the management of diabetes, the synthetic drugs play a crucial role but with critical side effects, which cover a range of conditions, including hypertension, atherosclerotic cardiovascular disease (ASCVD), endothelial cell dysfunction, nephropathy, dyslipidemia, hypercoagulopathy and retinopathy.^4^ Despite their clinical utility, synthetic antidiabetic agents are often associated with adverse effects and do not offer a comprehensive solution to the complex pathophysiology of T2DM.^5^

Regrettably, there are no universally effective treatments with fewer side effects available to manage the progression of diabetes. However, in recent years, numerous herbal medicines traditionally used to treat diabetes have demonstrated potential benefits for managing the disease, as evidenced by *in vivo* studies and clinical trials.^6^ The active phytochemical constituents found in medicinal plants and their products have been recognized as a valuable source of alternative medicine for the treatment of T2DM. These natural compounds may act individually or in combination, producing a synergistic effect that enhances their therapeutic benefits.^7^ In addition to their therapeutic benefits, medicinal plants offer several advantages over conventional pharmaceuticals, including safety profiles, good efficacy, wide availability, acceptability, and affordability. Given these advantages, medicinal plants have the potential to provide accessible and cost-effective healthcare solutions for a wide range of medical conditions.^8^

The growing popularity of traditional medicines in managing chronic diseases like T2DM is driven by their multitargeted actions, minimal side effects, and affordability.^9^ Herbal preparations have gained extensive recognition in India as curative agents, particularly those with cognitive-enhancing, blood sugar-lowering, liver-protective, and cholesterol-reducing properties.^10^ However, a major limitation of many herbal preparations is their poor dissolution profile, which directly impacts their bioavailability. The low solubility of active compounds often results in inadequate absorption, reducing the therapeutic efficacy of these formulations. This dissolution challenge is a critical barrier in the effective use of herbal medicines, particularly in conditions like T2DM, where consistent glucose regulation is crucial. Addressing this issue is vital for improving the clinical outcomes of herbal treatments.^11^

Polar compounds are generally expected to exhibit good aqueous solubility due to their ability to form hydrogen bonds with water molecules. This is because the presence of electronegative atoms such as oxygen and nitrogen, along with hydroxyl or carboxyl functional groups, enhances their interaction with the water. However, numerous polar compounds still exhibit poor aqueous solubility, largely due to factors such as high molecular rigidity, strong intramolecular hydrogen bonding, or tight crystalline packing.^12^ One such group of compounds are FCGs which represent a unique and pharmacologically significant class of dietary flavonoids that have garnered increasing scientific interest due to their broad spectrum of bioactivities. These compounds, naturally abundant in various medicinal plants, differ structurally from their more extensively studied O-glycoside counterparts and have shown notable antioxidant, anti-inflammatory, anticancer, hepatoprotective, and antidiabetic effects.^13^ Among them, flavone C-glycosides such as vitexin, isovitexin, orientin, and isoorientin are frequently reported for their potent biological actions. Despite their potential, FCGs especially monoglycosides exhibit poor intestinal absorption and are extensively degraded by gut microbiota, resulting in low systemic bioavailability.^14^ The previous study from our group isolated and identified 4 major bioactive FCGs namely Orientin, Isoorientin, Diosmetin and Luteolin from CM and they showed significant antidiabetic potential *in vitro* ^15^ Enhancing the solubility of these compounds is essential to ensure that the active compounds reach therapeutic concentrations at the target sites. Formulation development, using appropriate excipients or advanced drug delivery systems, is a widely accepted solution to this issue, as it improves dissolution and promotes better absorption of bioactive compounds.^16^ By addressing the solubility limitations through these formulation strategies, bioactive compounds can be effectively transformed into pharmacologically viable products with enhanced therapeutic potential.^17^

The development of an effective pharmaceutical formulation requires careful consideration of several critical factors, including the physicochemical and biopharmaceutical properties of the active pharmaceutical ingredient (API), such as solubility, stability, particle size, polymorphism, and p*K*_a_. In addition, the selection of suitable excipients, understanding potential API–excipient interactions, and optimizing the manufacturing process are vital to ensure product efficacy, stability, and patient compliance. These aspects are especially important when dealing with plant-based bioactives, which often present challenges such as poor solubility or stability.^18^ Tablet formulations, in particular, offer several advantages for improving the dissolution and overall efficacy of herbal extracts and bioactive compounds. Tablets provide a stable, precise, and easy-to-administer dosage form, which can enhance patient compliance. Moreover, modern tablet formulation techniques, can improve the solubility and dissolution rate of poorly soluble herbal compounds. These benefits make tablet formulations a highly attractive option for the delivery of herbal medicines.^19^ In recent years, several herbal formulations have been successfully developed and commercialized to enhance the bioavailability and therapeutic effect of plant-derived compounds. Formulations such as Diabecon from Himalaya and Epinsulin from Swastik formulations are known to enhance the peripheral use of glucose, support B cell repair, and mimic the actions of insulin.^20^ Moreover, polyherbal formulations like MD-1 have shown significant potential in improving glucose uptake and preventing adipogenesis in diabetes mellitus. The growing interest in natural medicinal drugs, especially a preference for herbal formulations, has led to the development of evidence-based alternative medicines using herbal preparations to manage diabetes effectively.^21^ Current studies are focused on active components of herbal formulations to enhance therapeutic efficacy and standardize products for better scientific results.

Notably, several studies suggest that C-glycosides may offer superior therapeutic benefits compared to O-glycosides and aglycones, particularly in the management of oxidative stress and metabolic disorders such as diabetes. However, *in vivo* evidence supporting these benefits remains limited, highlighting the need for further research to fully elucidate their pharmacokinetic properties and therapeutic potential.^13^ This research not only addresses the critical issue of poor dissolution in herbal drugs but also highlights the potential of formulation development to unlock new therapeutic possibilities for managing diabetes using natural products.

In this context, the current study focuses on the development of a scientifically optimized tablet formulation enriched with FCGs derived from CM. By overcoming solubility challenges through a wet granulation approach with optimized excipients, the study aims to enhance the dissolution, absorption, and ultimately the therapeutic efficacy of these bioactive FCGs in animal model. This approach not only provides a targeted solution to enhance bioavailability but also contributes to the growing body of evidence supporting the use of flavonoid-based phytopharmaceuticals in the effective management of T2DM.

## 2 Results and Discussion

### 2.1 Organoleptic and Physicochemical Characterization

The organoleptic and physicochemical properties of the plant material and its extracts are summarized in Table 1. Higher moisture content is known to accelerate decomposition rates and can significantly influence the overall characteristics of plant materials. Moisture content affects the cohesiveness of powder, impacting the formation of interparticle liquid bridges, which in turn promotes spontaneous particle agglomeration, leading to the formation of larger clusters.^22^ This phenomenon can have adverse effects on both extraction and formulation processes. However, the low moisture content of CM (8.5%) suggests minimal decomposition of the extract during formulation. The physicochemical analysis indicates that the material is well-suited for extraction and formulation purposes. The pH value of 6.5 ± 0.02 falls within a slightly acidic range, which is favorable for extracting active ingredients from the extract. This acidity level helps maintain the integrity and stability of active constituents, thereby enhancing the bioavailability of extracts.^23^ Furthermore, the low values of water-soluble ash (2.8 ± 0.11), acid-insoluble ash (0.80 ± 0.03), and total ash (3.12 ± 0.31) in the plant powder suggest a higher proportion of organic content compared to inorganic content. Overall, the organoleptic and physicochemical characteristics of the CM powder and CM-EA extracts support their suitability for formulation purposes.

**Table 1:** Organoleptic and Physicochemical Characterization of CM^a^

Physicochemical properties and Extractive value (CM)							Organoleptic Properties (CM-EA)			
TA (% w/w)	AIA (% w/w)	WSA (% w/w)	MC (% w/w)	pH at 25 °C	Water	Alcohol	Physical appearance	Color	Odor	Taste
3.12 ± 0.31	0.80 ± 0.03	2.8 ± 0.11	8.5 ± 1.02	6.5 ± 0.02	6.5 ± 0.58	10.2 ± 1.17	Small particulate	Dark brown	Aromatic	Mild sour

a CM, *Cassia mimosoides* powder; CM-EA, CM Ethyl acetate extract; TA, Total Ash; AIA, Acid insoluble ash; WSA, Water-soluble ash; MC, Moisture content.

### 2.2 Drug-Excipient Compatibility Test by FTIR Analysis

The infrared spectrum of CM-EA displayed several key absorption bands (Figure 1). A strong band at 3311.40 cm^–1^ indicates O–H stretching vibrations, likely from hydroxyl groups in secondary metabolites and residual moisture. C–H stretching vibrations are evident around 2924.10 cm^–1^. The presence of C = C double bonds is suggested by the band at 1606.41 cm^–1^, while bands at 1290.34 cm^–1^ and 1446.15 cm^–1^ point toward alkanes. Finally, a strong peak at 1018.78 cm^–1^ corresponds to C–O absorption. The various absorption bands observed in the CM-EA spectrum may arise from functional groups such as C = O, O–H, and C = C, which are present in alcohols and phenols. These functional groups are indicative of a variety of metabolites like tannins, flavonoids, anthraquinones, and others. The superimposed FTIR spectra obtained initially and after one month (Figure 2) showed no distinct changes in the banding patterns over time.

**Figure 1:**
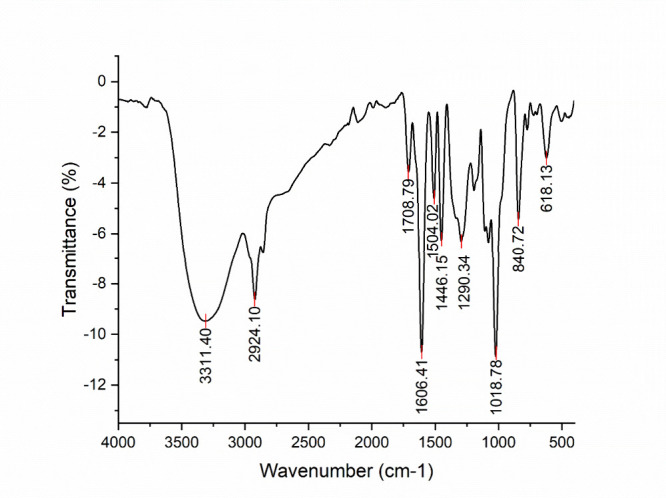
Infrared spectrum of the ethyl acetate extract of CM (CM-EA).

**Figure 2:**
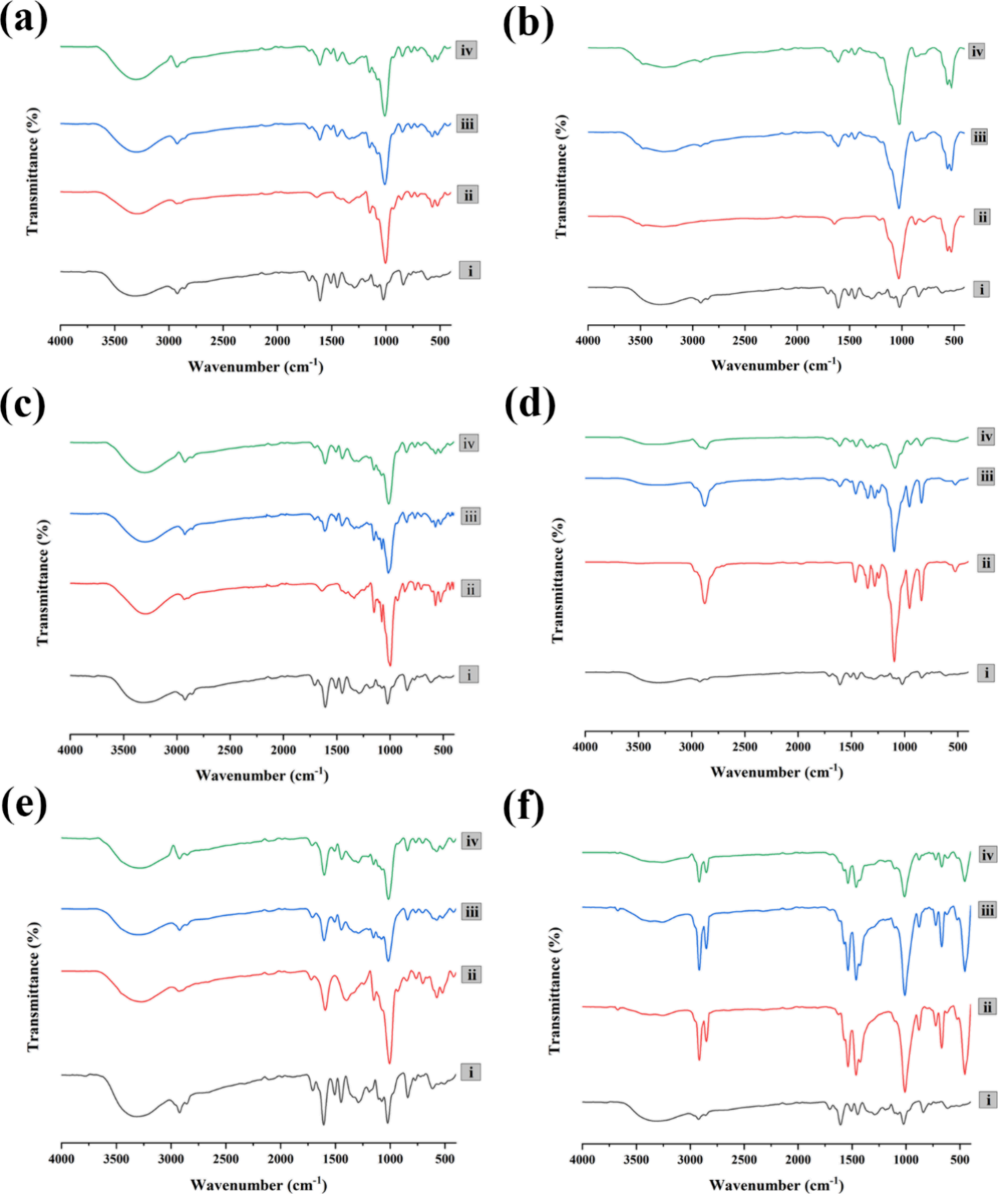
FTIR-ATR spectra of binary mixtures of CM-EA extract and pharmaceutical excipients. (a) i (CM-EA), ii (MCC), iii (CM-EA+MCC, BF), iv (CM-EA+MCC, AF); (b)) i (CM-EA), ii (DCP), iii (CM-EA+DCP, BF), iv (CM-EA+DCP, AF); (c)) i (CM-EA), ii (STH), iii (CM-EA+STH, BF), iv (CM-EA+STH, AF); (d) i (CM-EA), ii (PMR), iii (CM-EA+PMR, BF), iv (CM-EA+PMR, AF); (e) i (CM-EA), ii (SSG), iii (CM-EA+SSG, BF), iv (CM-EA+SSG, AF); (f) i (CM-EA), ii (MSE), iii (CM-EA+MSE, BF), iv (CM-EA+MSE, AF); (BF-Before one month, AF-After one month)

### 2.3 Flow Properties

In accordance with the standard reference values set by the United States Pharmacopeia (USP), it can be observed that the batch CM F4 demonstrates a relatively lower value (13.79 ± 0.49) for the compressibility index when compared to the remaining batches (CM F1 to CM F3). This observation implies that the granules utilized in CM F4 display superior flow properties (Figure 3 and Supplementary File, Table S1) and a reduced propensity for cohesive agglomeration during tablet compression.^24^ Formulation batch F4 demonstrated excellent flowability and consolidation properties compared to other batches, as evidenced by superior Hausner ratios (1.16 ± 0.02) and reduced angles of repose (32.14 ± 0.98). The improved Carr indexes indicate a lower difference between tapped and bulk densities, resulting in better flowability. Additionally, the superior Hausner ratios suggest improved compaction and densification during tableting, leading to uniform tablet hardness and reduced risk of defects. The lower angles of repose further support the enhanced flowability of granules in batch F4.^25,26^ These findings emphasize the importance of optimized excipient selection and manufacturing parameters in achieving desirable granule characteristics for efficient pharmaceutical processing and tableting.

**Figure 3:**
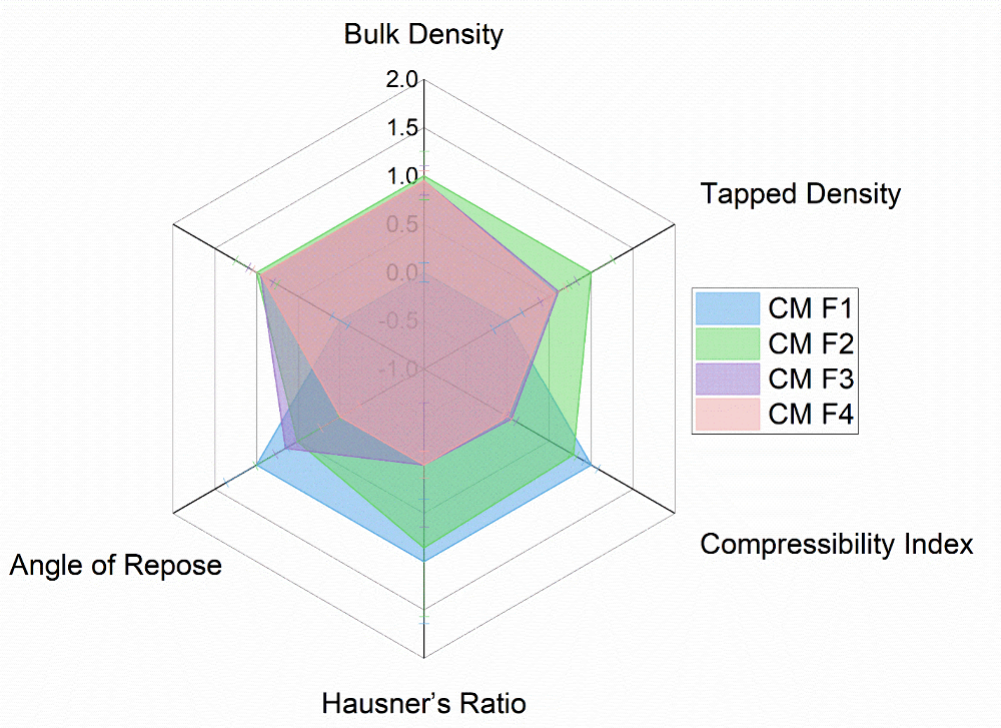
Radar chart illustrating the normalized flow properties of four tablet formulations (CM F1–F4). Each axis represents a distinct flow property: Bulk Density, Tapped Density, Compressibility Index, Hausner’s Ratio, and Angle of Repose. Data were normalized using min–max scaling (0–1 range) without inversion, such that higher normalized values represent higher absolute values. This chart allows visual comparison of flow behavior across formulations, where tighter and larger polygons suggest better flow-related characteristics depending on the original directionality of each parameter. Values are expressed as the mean ± SD of triplicate values.

### 2.4 Quality Assessment of the Tablets

The result of weight variation, hardness, and friability are represented in Table 2. The CM F4 batch of tablets demonstrated superior quality and performance, distinguished by its optimal weight uniformity, desirable hardness, and minimal friability when compared with the other batches. Weight variation is a critical parameter reflecting the consistency in tablet weight within a batch. This characteristic is vital for ensuring the tablets can withstand external forces without damage, maintaining their integrity during handling and transportation.^27^ In the case of CM F4, the weight variation is minimal (501 ± 0.75), indicating high precision during the manufacturing process. This is a crucial factor as it ensures that each tablet contains the intended dosage of the herbal ingredient. Moreover, the ability of a tablet to endure mechanical stress while being handled and transported without disintegration or fracture is determined by its hardness. In a study, an oral tablet formulation using Wuzi Yanzong dry extract (WYE) was developed to tackle challenges associated with high drug loadings and suboptimal physicochemical and mechanical properties of dry herbal extracts. Critical quality attributes were identified as tablet disintegration time (DT) and hardness.^28^ Similarly, when compared to other batches, the hardness of CM F4 tablets is notably greater (7.64 ± 0.29), signifying a robust composition and compression procedure. This attribute is essential as it ensures that the tablets remain intact throughout their shelf life, maintaining their structural integrity and preventing any loss of potency. Tablet friability refers to the inclination of a tablet to experience the separation of constituent particles due to abrasion, friction, or mechanical impact. The friability of CM F4 tablets is remarkably low compared to other batches, indicating excellent tablet strength and resistance to breakage. This attribute ensures that the tablets can withstand normal handling during packaging, transportation, and consumption without significant loss or degradation of the active herbal ingredients.

**Table 2:** Post-compression Study Data of Compressed Tablets^a^

Formulation	Hardness (Kgf)	Friability (%)	Weight variation (%)	Disintegration (DT-min)
CM F1	6.90 ± 0.85	0.43 ± 0.02	502.0 ± 3.12	12.0 ± 1.11
CM F2	7.64 ± 0.29	0.65 ± 0.03	500.5 ± 2.06	0.58 ± 0.12
CM F3	6.70 ± 0.38	0.88 ± 0.05	504.0 ± 1.69	1.16 ± 0.21
CM F4	8.30 ± 0.55	0.26 ± 0.01	501.0 ± 1.32	0.75 ± 0.16

a DT values in seconds were converted and represented in minutes. Values are expressed as the mean ± SD of triplicate values.

### 2.5 *In Vitro* Dissolution and Disintegration Tests for the Formulated Tablets

In a study conducted by Xin and Zheng, the disintegration test was used to optimize the shaping technology and content determination of the traditional herbal formula Genhuang dispersible tablets. The results showed that the prepared dispersible tablets were able to disintegrate completely within 3 min, meeting the standards of Chinese pharmacopeia.^29^ Remarkably, the DT results of the CM F4 tablet batch demonstrated a rapid disintegration time of 45 s (0.75 min), as depicted in Table 2. The quick DT of the CM F4 tablet batch observed in this study can be attributed to several factors, including the selection and compatibility of excipients, particle size, hardness, and optimal moisture content. An essential component contributing to the rapid disintegration of the CM F4 tablet batch is the incorporation of SSG as the active disintegrant in the formulation. SSG has been widely recognized for its effectiveness in initiating the disintegration process of tablets. Studies have shown that the quality of disintegrants and their compatibility with the API are crucial factors that can significantly impact drug disintegration rate and bioavailability.^30^ The dissolution test serves as a vital tool for assessing the quality of herbal medicines in solid dosage forms intended for oral use. A dissolution study conducted using tablets formulated with *Ximenia americana* L. extract revealed that approximately 70% of the vegetable extract content was released within 30 min. Utilizing the direct compression method for producing herbal tablets facilitated a rapid formulation and production process, ensuring compliance with quality standards.^31^ In comparison, the CM F4 batch gives a 52% release of API within 30 min and is extended to 90% in 2 h (Figure 4). This particular formulation batch demonstrated a more rapid release of the API compared to other batches. Another study investigated the solubility of *Curcuma comosa* oleoresin-like crude extract tablets using the liquid solidification method. *In vivo* pharmacokinetic studies showed that the optimized liquid-soluble formulations retained the same properties, had good drug properties, and exhibited improved dissolution behavior.^32^ These factors collectively contribute to the enhanced disintegration and dissolution of the tablets made from CM-EA during formulation development and are critical considerations in formulating effective herbal tablets.

**Figure 4:**
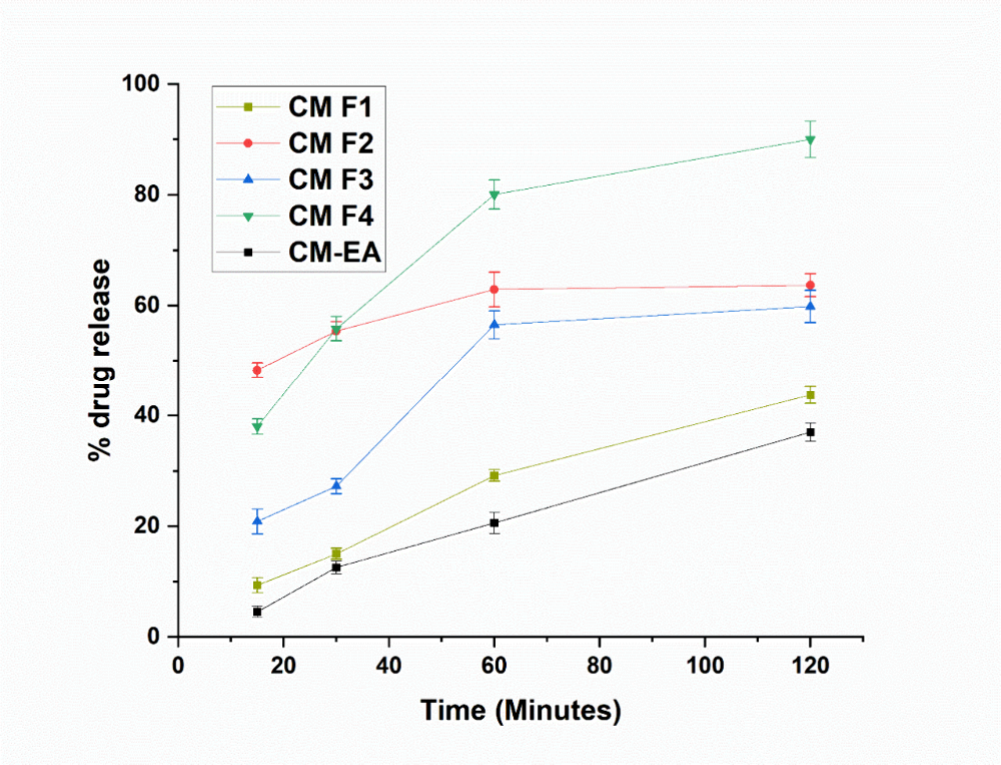
*In vitro* dissolution profile of different formulation batches (CM F1-CM F4) using CM-EA as API in a medium containing 0.1 M HCL (mean ± SD, *n* = 3)

### 2.6 HPLC Quantification of Bioactive FCGs in Herbal Tablets

Importantly, our analysis confirmed the stability and integrity of the four previously isolated bioactive FCGs such as orientin, isoorientin, diosmetin, and luteolin within the CM F4 tablets (Figure 5, Table 3, Supplementary File, Table S2). The quantification revealed negligible variation in their amounts compared to the extract CM-EA used for tablet preparation. This finding is crucial as it suggests that the identified bioactive FCGs remain stable throughout the formulation process and are likely contributing to the observed antidiabetic effects. Orientin and isoorientin, have been reported to exhibit various pharmacological activities, including antidiabetic effects. Studies have shown that orientin can improve insulin sensitivity, as evidenced by its ability to reduce insulin resistance induced by 2,3,7,8-tetrachlorodibenzo-p-dioxin in adipocytes.^33^ Additionally, orientin exhibits a protective effect on pancreatic β-cells against oxidative stress and lipotoxicity.^34^ Similarly, isoorientin demonstrates hypoglycemic effects, potentially by enhancing glucose uptake and positively influencing lipid metabolism.^35,36^ Diosmetin, a flavone aglycone, has also garnered attention for its antidiabetic potential. Research suggests that diosmetin can improve glucose uptake,^37,38^ reduce insulin resistance, and protect against diabetic nephropathy.^39^ Luteolin, another flavone present in CM F4, possesses potent anti-inflammatory and antioxidant properties.^40,41^ These properties are particularly relevant in the context of diabetes, as chronic inflammation and oxidative stress are recognized as significant contributors to disease progression and the development of complications.^42^ The presence and stability of these bioactive FCGs in CM F4, coupled with their documented antidiabetic properties, strongly suggest their synergistic contribution to the observed therapeutic efficacy. The structure of the isolated compounds were represented in Figure 6.

**Figure 5:**
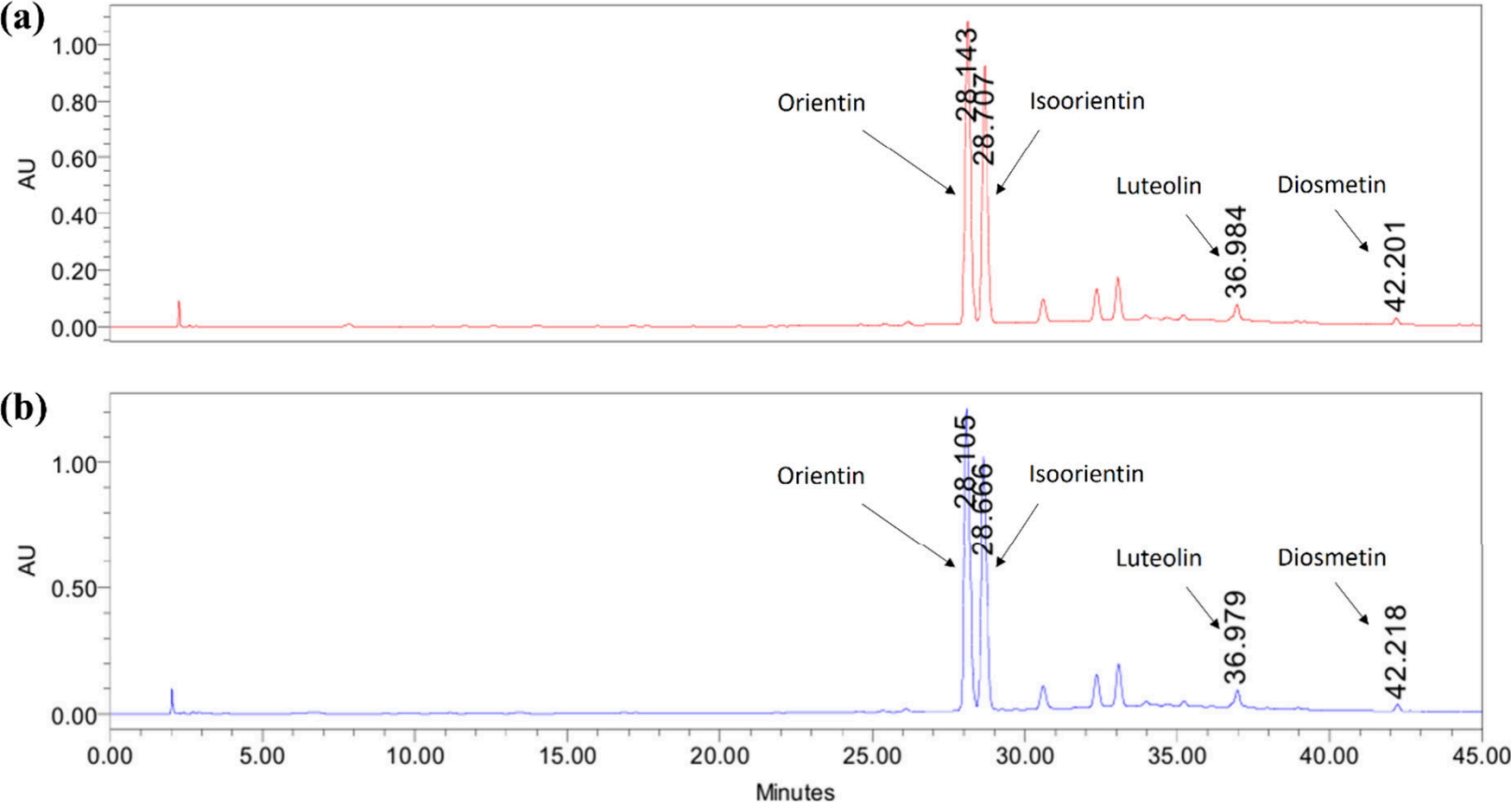
Comparative HPLC estimation of bioactive FCGs. (a) Chromatogram representing the bioactive FCGs orientin, isoorientin, luteolin, and diosmetin with retention time in CM F4 tablet. (b) Chromatogram representing the bioactive FCGs in CM-EA (API).

**Table 3:** Bioactive FCGs Estimation with Retention Time from CM F4 and CM-EA^a^

	RT (min)		Quantity in mg/g of CM-EA extract or tablet		
Bioactive FCGs	CM-EA	CM F4	CM-EA	CM F4	Relative % of actives in tablet against extract
Orientin	28.105	28.143	101.70 ± 1.04	99.34 ± 0.28	99.16 ± 0.82
Isoorientin	28.666	28.707	110.24 ± 0.97	109.3 ± 0.55	95.27 ± 0.99
Luteolin	36.979	36.984	4.99 ± 0.33	4.75 ± 0.09	95.25 ± 0.71
Diosmetin	42.218	42.201	1.14 ± 0.04	1.09 ± 0.05	97.67 ± 0.18

a The values are presented as mean ± SD, derived from triplicate measurements performed over 3 different runs.

**Figure 6:**
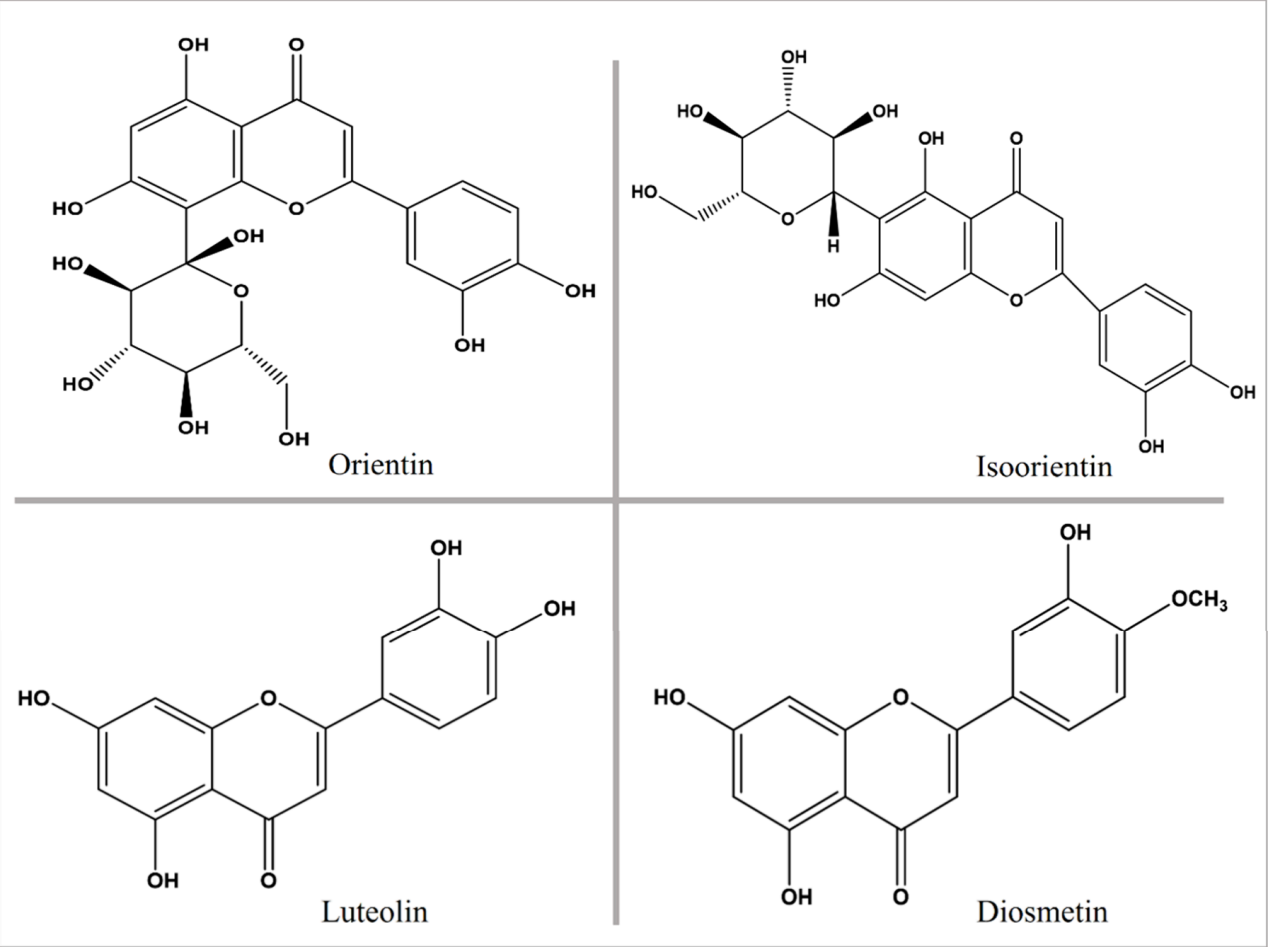
Chemical structures of the major bioactive FCGs identified from CM: Orientin, Isoorientin, Luteolin, and Diosmetin.

### 2.7 Estimation of Body Weight, Blood Glucose Level, and OGTT

High-fat diets are known to promote weight gain, impaired glucose tolerance, and insulin resistance, which are among the primary risk factors for developing T2DM.^43^ In our study, the HFD–STZ protocol successfully induced T2DM in rats by combining a calorie-dense diet with partial β-cell dysfunction induced by low-dose STZ. The formulated diet (4225 kcal/kg; ∼ 51% calories from fat) resulted in a rapid body weight increase from ∼ 250 g to ∼ 350 g within 3 weeks, consistent with earlier reports demonstrating that energy-dense diets can induce insulin resistance within 2–3 weeks.^44–46^ The treatment groups exhibited significantly higher weight gain compared to the normal control group during the HFD period. Thus, our model offers a time-efficient, cost-effective, and reproducible approach to T2DM induction. While this model effectively reproduces the dual pathology of T2DM. After administering STZ, the treatment groups, including the diabetic control, experienced a rapid increase in blood glucose levels and a significant decrease in body weight. Additionally, their food and water intake exceeded that of the normal control group. The condition was observed to improve upon treatment with a dose of 75 and 150 mg/kg of the CM F4 formulation batch. However, the CM F4–150 batch showed a promising improvement in all the parameters analyzed than the CM F4–75 batch. The group treated with CM F4–150 showed a significant halt in weight reduction, approximately 250 g, which closely approximated the initial weight of the control group rats (*p* < 0.05) (Supplementary File, Table S3). The restoration in weight may be due to the protective effect of the CM extract on structural proteins, which prevented their degradation.^47^ The diabetic group demonstrated a marked rise in fasting blood glucose levels (318.14 ± 9.27 to 451.01 ± 17.97 mg/dL) due to the persistent damage of β cells induced by STZ. The metformin and CM F4 treatment groups demonstrated a significant (*p* < 0.05) dose-dependent reduction in blood glucose levels throughout the experimental period compared to untreated diabetic rats. The study found that the highest dose (150 mg/kg) of CM F4 was able to decrease blood sugar at a rate that was higher than the standard drug metformin (CM F4–150 – 490.98 ± 14.52 to 119.64 ± 9.45, Met – 489.01 ± 16.28 to 129.59 ± 8.77) (Figure 7) (Supplementary File, Table S4). The potential reason behind this could be the induction of escalated secretion of insulin from the pre-existing cells, regenerated pancreatic beta cells and the improved cell sensitivity.^48,49^ The histopathological analysis of the pancreas also supports the confirmation of these observations. Natural remedies derived from medicinal plants demonstrate their efficacy in combating hyperglycemia by effectively addressing underlying causes. This is achieved through multiple mechanisms, including the inhibition of free radical generation, prevention of nonenzymatic protein glycation, reduction of glucose metabolism via the hexosamine pathway, inhibition of the activation of the polyol pathway, blockade of protein kinase C activation mediated by glucose, and minimization of lipid peroxidation.^50^ In agreement with the above reports, the proven capability of CM active constituents will synergistically collaborate within CM F4, resulting in the amelioration of the hyperglycemic condition in the treated groups. A study investigated the antidiabetic properties of *Gastrodia elata* Blume water extract in a rat model of diet-induced type 2 diabetes. The extract, administered at concentrations of 0.5% and 2% for 8 weeks, significantly improved glucose tolerance and reduced hepatic glucose production.^51^ In our study, on the final day, an oral glucose tolerance test (OGTT) was conducted to evaluate the insulin-sensitizing abilities of CM F4. Both the CM F4–150 and metformin-treated groups exhibited a dose-dependent improvement in OGTT at all-time points compared to the diabetic control group (Figure 8) (Supplementary File, Table S5). This suggests that treatment with CM F4–150 effectively manages insulin resistance and enhances glucose metabolism.

**Figure 7:**
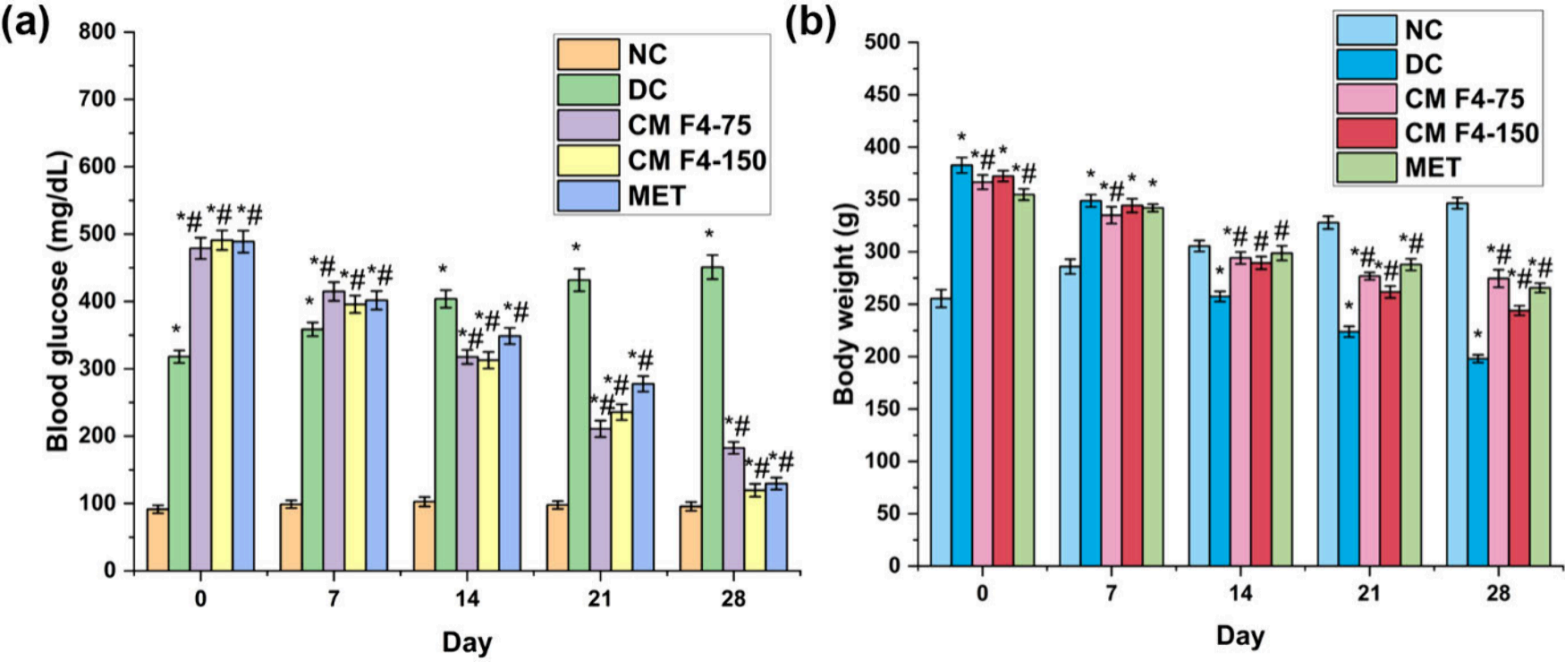
Effect of CM F4 formulation on (a) blood glucose level and (b) body weight in HFD-STZ-induced type 2 diabetic Sprague–Dawley rats. The values are presented as mean ± SD (*n* = 6). *p < 0.05 indicates significance compared to the NC group, while ^#^ *p* < 0.05 denotes significance compared to the DC group. NC, normal control; DC, diabetic control; CM F4–75, diabetic rats + formulation batch 4 (75 mg kg^–1^); CM F4–150, diabetic rats + formulation batch 4 (150 mg kg^–1^); MET, diabetic rats + metformin (100 mg kg^–1^)

**Figure 8:**
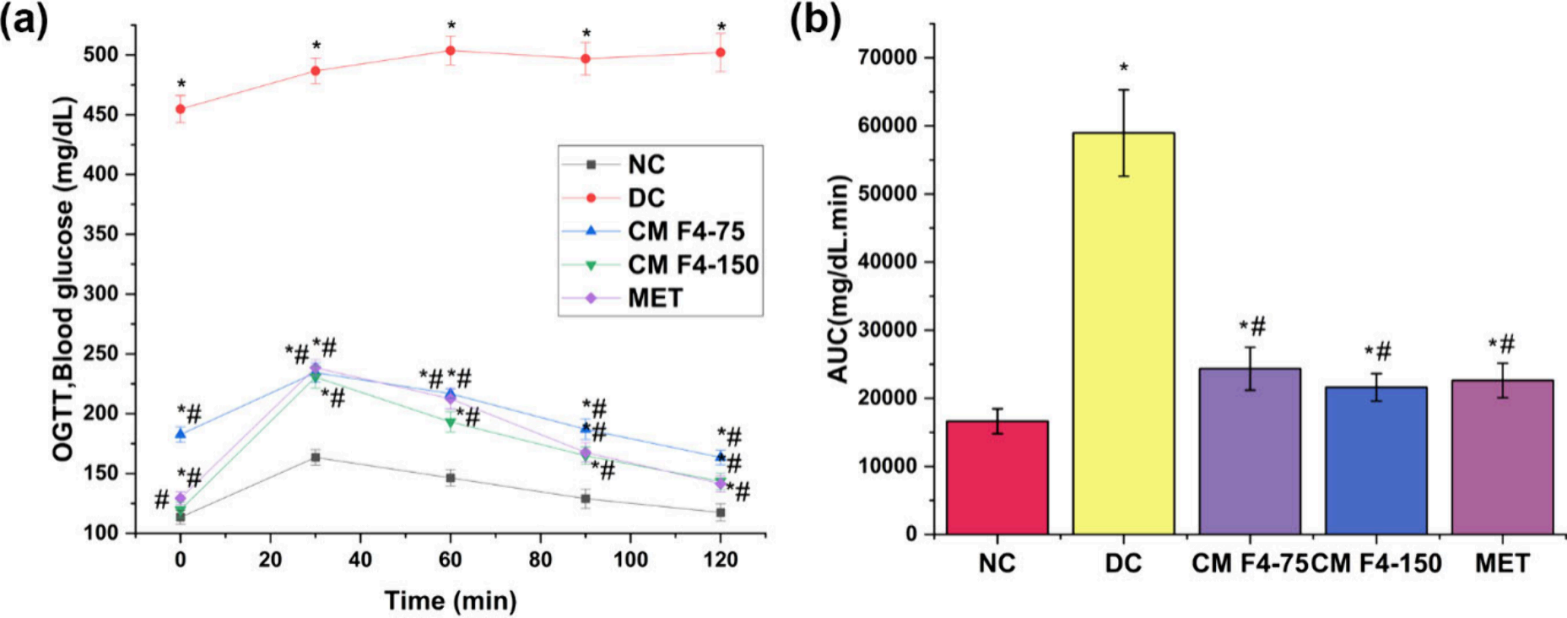
Effect of CM F4 formulation in HFD-STZ-induced type 2 diabetic Sprague–Dawley rats on (a) OGTT blood glucose levels; (b) OGTT area under the curve (AUC_0–120_ min), after administration for 28 days. The values are expressed as mean ± SD (*n* = 6). *p < 0.05, as compared to NC group. ^#^ *p* < 0.05, as compared to DC group. NC, normal control; DC, diabetic control; CM F4–75, diabetic rats + formulation batch 4 (75 mg kg^–1^); CM F4–150, diabetic rats + formulation batch 4 (150 mg kg^–1^); MET, diabetic rats + metformin (100 mg kg^–1^)

### 2.8 Insulin Resistance and β-Cell Function

In order to assess the levels of insulin sensitivity and resistance among different treatment groups, the most extensively validated mathematical models in both rodents and humans, like HOMA-IR, HOMA-β, and QUICKI, were employed.^52^ The OGTT findings were further supported by the results for indices of β–cell function and insulin resistance. The HOMA-IR values were significantly (*p* < 0.05) reduced in rat groups treated with CM F4–150 (5.74 ± 0.73) compared to the diabetic control (11.76 ± 1.31). This indicates the ability of the formulation to improve insulin sensitivity, which in turn helps to clear up hyperglycemia. The enhanced sensitivity of the cells was confirmed through the validation of the QUICKI index results. The decrease in HOMA-β value observed in the diabetic control group (4.93 ± 1.39) unequivocally indicates the impaired performance of the β cells.^53^ Conversely, the group administered with CM F4–150 successfully restored the functionality of these cells (51.26 ± 4.61) to a normal state (Figure 9). Several herbal products have shown promising results in improving β-cell function, as indicated by HOMA-β. Investigations have provided evidence of substantial regeneration of pancreatic β-cells, characterized by an augmentation in the size and number of pancreatic islets, along with an increase in the number of insulin-secreting cells and proliferating β-cells, as determined by histopathological and immunohistochemical evaluations.^54,55^

**Figure 9:**
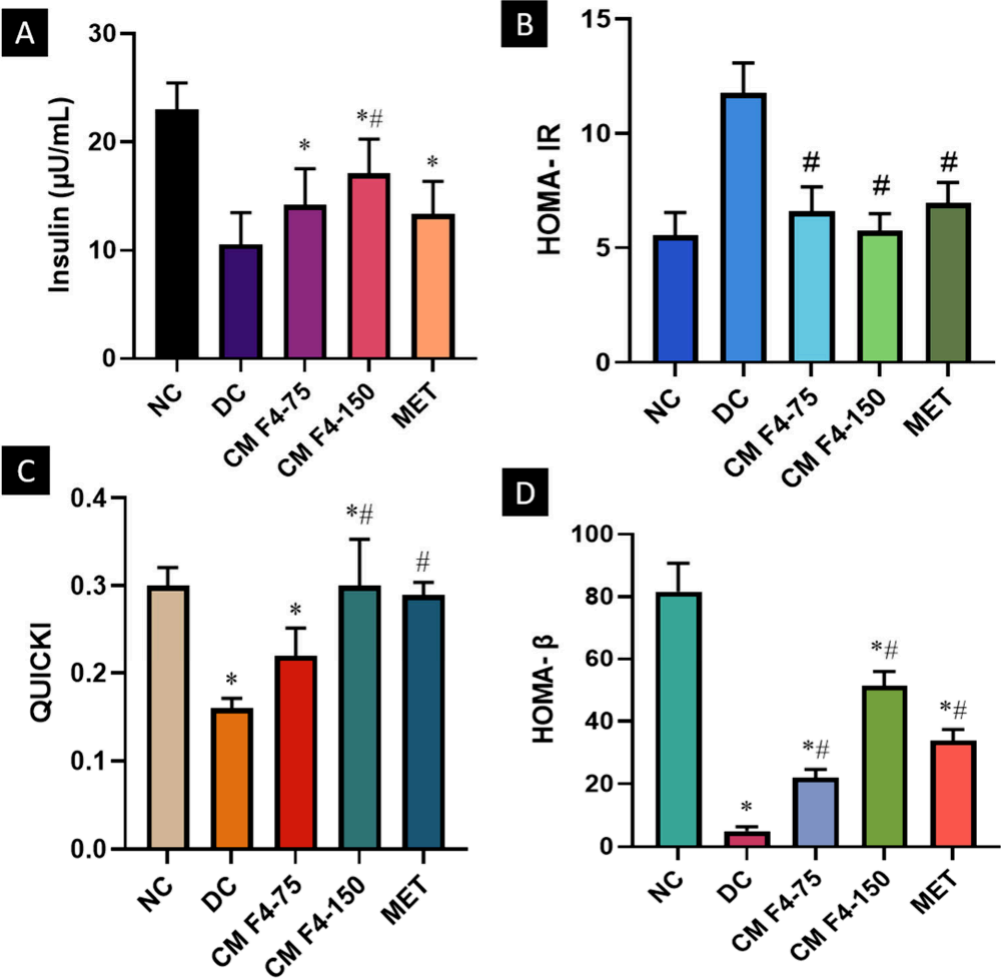
Effect of CM F4 formulation on (a) plasma insulin level, (b) the statistical models HOMA- IR (c) QUICKI, and (d) HOMA-β in HFD-STZ-induced type 2 diabetic Sprague–Dawley rats. All values are expressed as mean ± SD (*n* = 6). Significance was accepted at *p < 0.05, ^*#*^ *p <*0.05, *Statistical significance compared to the NC group; ^#^statistical significance compared to the DC group.

Moreover, the CM F4–150 group displayed a notable increase in serum insulin concentration, suggesting the potential rejuvenation or restoration of pancreatic cells, possibly mediated through the antioxidant properties of the CM extract. Similar findings have been reported in various studies using different plant extracts. For instance, the acetone fraction from the fruit extract of *Xylopia aethiopica* and the aqueous extract of *Aegle marmelos* fruit and leaves in an HFD/STZ-induced T2DM rats model supported the results mentioned above.^56,57^ In addition to mitigating various biochemical and hematological changes, the treatment with CM F4 demonstrated the ability to reverse resistance to insulin and facilitate the repair or regeneration of pancreatic beta cells. Histopathological findings from the pancreas further supported the results obtained from these mathematical models.

### 2.9 Hepatic and Renal Markers

The kidneys and liver are vital organs that come into contact with diverse toxins and metabolic byproducts within the body. Therefore, monitoring changes in hepatic and renal markers can provide valuable insights into the effects of various drugs being tested, allowing for a better understanding of their impact on these organs.^58^ Diabetes is commonly associated with increased levels of liver toxicity marker enzymes, including ALP, AST, ALT, and GGT, in the bloodstream. This is primarily due to the release of these enzymes from the liver cytosol into circulation. Besides, the disruption in metabolic processes and abnormalities in kidney function leads to an elevation in the levels of urea, uric acid, and creatinine within the bloodstream.^59,60^ According to the above reports, the activity of such enzymes and byproducts significantly increased in the diabetic control group, indicating damage to the liver and kidney. The dosage of CM F4–150 in diabetic rats significantly (*p* < 0.05) reduced the toxicity markers in blood serum, conveying its protective effect in administration (Table 4). This could be due to the potential of the formulation to enhance the regeneration of liver cells and restore kidney functioning, leading to a decrease in the release of marker enzymes and byproducts into the bloodstream of the rats.^61^ The outcomes were also supported by prior studies proving the hepato-renal protective effects of ethanol extracts from *Citrullus colocynthis* and *Momordica charantia* in male albino rats with STZ-induced diabetes.^62^

**Table 4:** Hepatic and Renal Toxicity Markers^a^

Group	SGPT/ALT (IU/L)	SGOT/AST (IU/L)	GGT (IU/L)	ALP (IU/L)	Urea (mg/dL)	Uric acid (mg/dL)	Creatinine (mg/dL)
NC	33.95 ± 4.52	84.83 ± 7.80	18.99 ± 4.54	118.04 ± 6.45	22.47 ± 1.85	3.34 ± 0.97	0.59 ± 0.13
DC	85.46 ± 4.44^b^	124.64 ± 9.26^b^	39.61 ± 3.76^b^	194.89 ± 10.16^b^	38.49 ± 2.61^b^	9.98 ± 2.79	0.99 ± 0.05^b^
CM F4–75	51.75 ± 6.04^b^ ^c^	113.40 ± 7.16^b^ ^c^	28.00 ± 5.79^b^ ^c^	158.95 ± 7.19^b^ ^c^	28.32 ± 3.46^b^ ^c^	6.47 ± 3.18^b^	0.72 ± 0.16^c^
CM F4–150	40.87 ± 4.63^c^	91.54 ± 6.07^b^ ^c^	22.73 ± 4.97^c^	129.54 ± 6.53^c^	26.5 ± 3.54^c^	4.19 ± 2.13	0.62 ± 0.05
Met	42.91 ± 5.09^b^ ^c^	88.54 ± 5.14^b^ ^c^	20.81 ± 3.67^c^	125.00 ± 7.00^c^	25.33 ± 3.77^c^	5.68 ± 1.46^c^	0.72 ± 0.20^c^

a Effect of CM F4 formulation on Hepatic and Renal toxicity markers in HFD-STZ-induced T2DM rats. Values are expressed as mean ± SD (*n* = 6).

b Statistically significant compared to normal control.

c Statistically significant compared to diabetic control. Significance accepted at *p* < 0.05.

### 2.10 Carbohydrate Metabolizing Enzymes

Insulin exerts its influence on the intracellular utilization of glucose through various mechanisms. It stimulates the activation of enzymes involved in glycolysis while simultaneously inhibiting the activity of enzymes involved in gluconeogenesis, thereby ensuring the postprandial regulation of blood glucose levels. In instances of insulin deficiency and resistance, which are prominent characteristics of diabetes, there is a disruption in the regulation of carbohydrate metabolism and a decline in the peripheral utilization of glucose, leading to an increase in hepatic glucose production.^63^ As an insulin-sensitive enzyme and a gateway to glycolysis, hexokinase function will be halted in the absence or low amount of insulin in HFD-STZ-induced diabetic rats.^64^ Pyruvate kinase (PK), another regulatory enzyme in glycolysis, facilitates the conversion of phosphoenol pyruvate into pyruvate, releasing ATP. PK dysfunction leads to the buildup of glycolytic intermediates, particularly 2,3-bisphosphoglycerate (2,3-BPG), which hinders the flow of glycolysis by inhibiting hexokinase.^65^ The current investigation revealed the decreased activity of these enzymes in the hepatic tissue of rats with HFD-STZ induced diabetes. When CM F4 was given orally to diabetic rats at a dose of 150, the activity of the aforementioned enzymes was substantially restored to nearly normal levels (Figure 10). Our results are in line with Gothandam et al., who reported that treatment with theaflavin in HFD-STZ diabetic rats reestablished altered activities of the above enzymes into near normal in liver and kidney tissues.^66^ In the pathophysiology of diabetes mellitus, the increased endogenous production of glucose is predominantly attributed to increased gluconeogenesis and glycogenolysis. The deficiency of insulin and an abundance of gluconeogenic substrates activate these enzymes, thereby disturbing glucose homeostasis and resulting in hyperglycemia.^67^ This study found elevated glucose-6-phosphatase and fructose-1,6-bisphosphatase activity in the hepatic tissues of diabetic rats compared to the control group. The formulation CM F4–150 demonstrated effective inhibition of gluconeogenic enzymes, regulating their levels to a near-normal state and exhibiting a similar activity to metformin. Supporting results were obtained from the studies carried out by Kurup et al. and Alruhaimi et al. in HFD-STZ and STZ-induced diabetic rats using a fraction rich in flavonoids obtained from the ethyl acetate extract of *Euphorbia peplus* and the ethyl acetate extract of *Averrhoa bilimbi* fruit.^68,69^ In summary, CM F4 has improved peripheral glucose utilization by reestablishing altered levels of glycolytic and gluconeogenic enzymes, indicating its potential for managing hyperglycemia.

**Figure 10:**
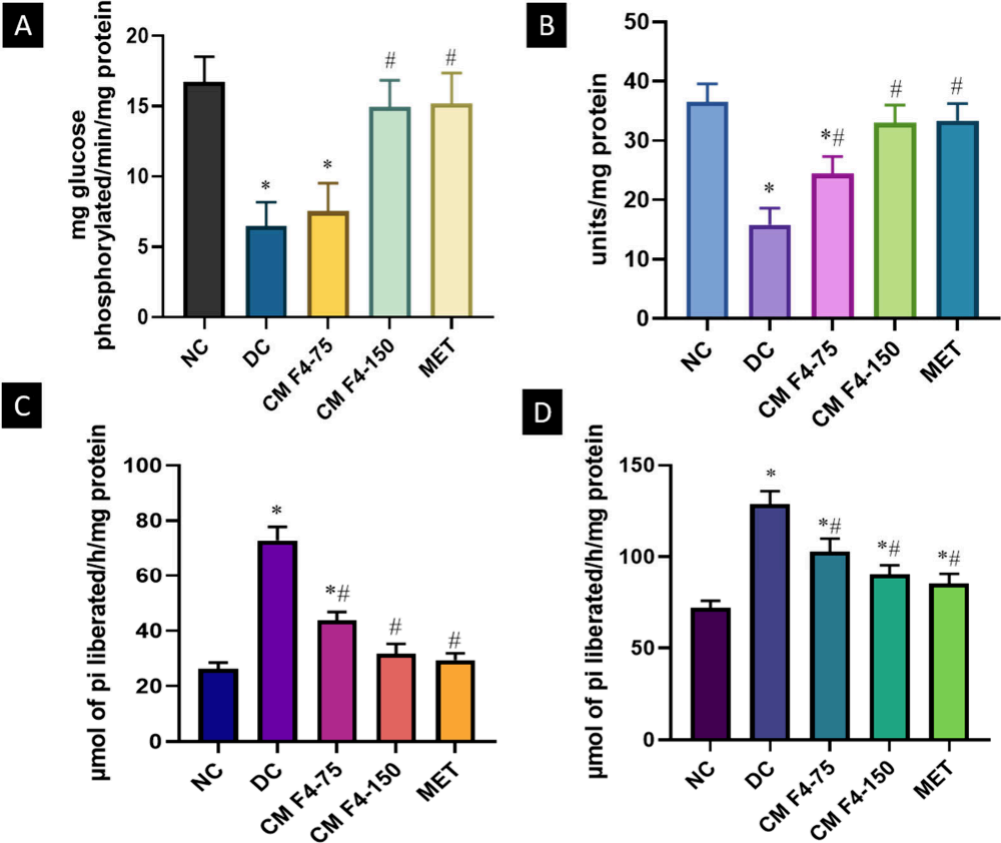
Effect of CM F4 formulation on carbohydrate metabolizing enzymes (a) Hexokinase, (b) Pyruvate kinase, (c) Glucose-6-phosphatase (d) Fructose 1,6-bis-phosphatase in HFD-STZ-induced type 2 diabetic Sprague–Dawley rats. All values are expressed as mean ± SD (*n* = 6). Significance were accepted at *p* < 0.05. *Statistically significant as compared to the NC group. ^#^Statistically significant as compared to the DC group.

### 2.11 Histopathology of Pancreas

Histopathological observations revealed apparent pancreatic islet atrophy in diabetic rats, characterized by reduced islet size and number, loss of structural integrity of islets, and moderate levels of inflammation. However, diabetic rats treated with CM F4, particularly at a dose of 150, reversed atrophy and improved cell number and size (Figure 11). These changes indicate the regenerative potential and safety profile of the CM F4 tablet. The study conducted by Satyanarayana supports our findings, demonstrating that *Solanum torvum* fruit extract elicited an increase in the number, size, and regeneration of β-cells within the islets of Langerhans in streptozotocin-induced diabetic rats.^70^

**Figure 11:**
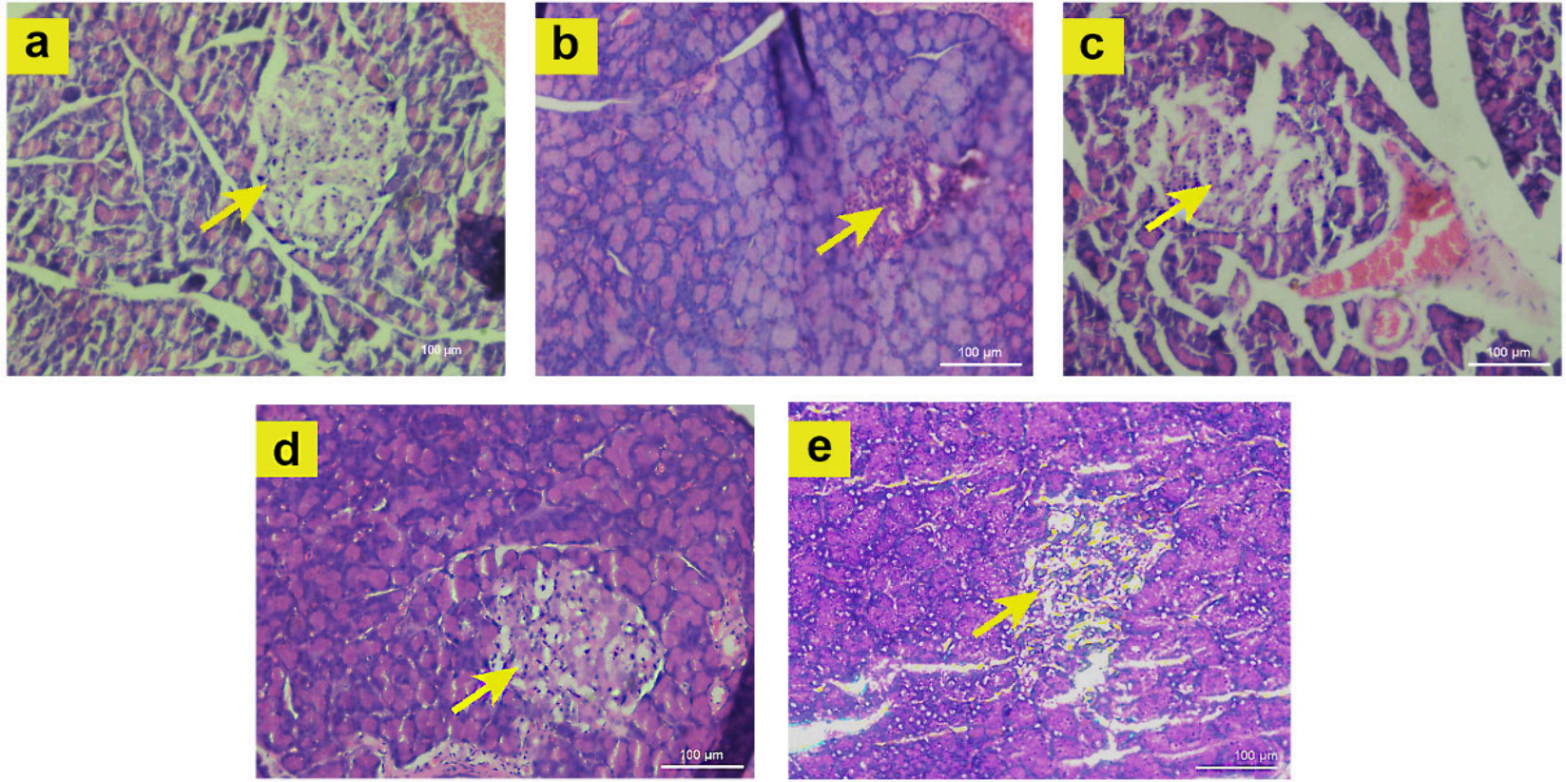
Histopathological analysis of pancreatic tissue from HFD-STZ induced T2DM rats treated with CM F4 formulation is presented as follows: (a) Nondiabetic Control – arrow denotes normal proliferating pancreatic islets, (b) Diabetic Control – arrow indicates degenerated and shrunken pancreatic islets, (c) CM F4–75 treated group – arrow denotes partial recovery of pancreatic islets, (d) CM F4–150 treated group – arrow denotes 90% recovery of pancreatic islets, (e) Metformin treated group – arrow shows a slight recovery of islets following disease-induced destruction.

### 2.12 Effect of CM F4 on mRNA Expression

Approximately 80% of whole-body glucose metabolism is attributed to skeletal muscle activity. GLUT4 is a crucial glucose transporter and acts as the primary mediator of insulin-stimulated glucose uptake in insulin-responsive tissues, especially in skeletal muscle. Herein, the translocation of GLUT4 is an essential step in enhancing glucose uptake.^71^ This is achieved mainly through major pathways such as insulin-dependent PI3K/Akt mediated pathway and insulin-independent AMPK (AMP-activated protein kinase) mediated pathway.^72^ The insulin signaling pathway in skeletal muscle is disrupted in HFD-STZ type 2 diabetes mellitus. This disruption is characterized by increased serine phosphorylation of IRS1, reduced AKT phosphorylation, and impaired GLUT4 translocation to the cell membrane.^73^ The energy imbalance (ATP/AMP ratio) leads to the activation of the sensor AMPK and is mediated by various factors like intracellular concentrations of calcium and bradykinin, activation of mitogen-activated protein kinase (MAPK), and activation of Ca^(2+)^/calmodulin-dependent protein kinase (CaMK).^74^ Ultimately, these impairments in the signaling cascade of glucose metabolism lead to the hallmark characteristic of reduced glucose uptake in skeletal muscle, a crucial feature of T2DM pathophysiology. To understand the molecular mechanism underlying the effects of the CM F4 formulation on glucose clearance, mRNA expression studies were carried out. In the diabetic group, the mRNA expression levels of genes involved in both insulin-independent and insulin-dependent pathways were significantly reduced. Treatment with CM F4 and metformin, however, led to a marked improvement in gene expression levels (*p* < 0.05). The group treated with CM F4 at a dose of 150 mg/kg demonstrated an upregulation of mRNA expression levels comparable to those observed in the metformin-treated group (Figure 12) (Supplementary File, Tables S6–S10). In both the pathways, the key genes such as IRS-1, PI3K, Akt, GLUT4, and AMPK were significantly upregulated and together enhanced the translocation of GLUT4 for improved glucose metabolism. In a study conducted by Mishra et al., 2023 reported that the compound tinosporaside from *Tinospora cordifolia* were showed an upregulation in mRNA expression of Both PI-3-Kinase- and AMPK-dependent pathway genes in diabetic db/db mice model. An improved GLUT4 expression in muscle tissue also resulted from a study in which mustard oil were administered to alleviate the STZ induced diabetes in rats.^76^ Moreover, a study investigating two derivatives of embelin, namely 6-bromoembelin and vilangin, in HFD-STZ induced T2DM rats, demonstrated increased insulin-mediated glucose uptake. These compounds were found to upregulate PPARγ expression and function as partial PPARγ agonists by promoting GLUT4 translocation, thereby stimulating glucose transport through the PI3K/Akt signaling cascade. These findings were further supported by molecular docking results.^77^ This supporting information substantiates the effect of CM F4 in improving the expression of both PI3K and AMPK-associated genes.

**Figure 12:**
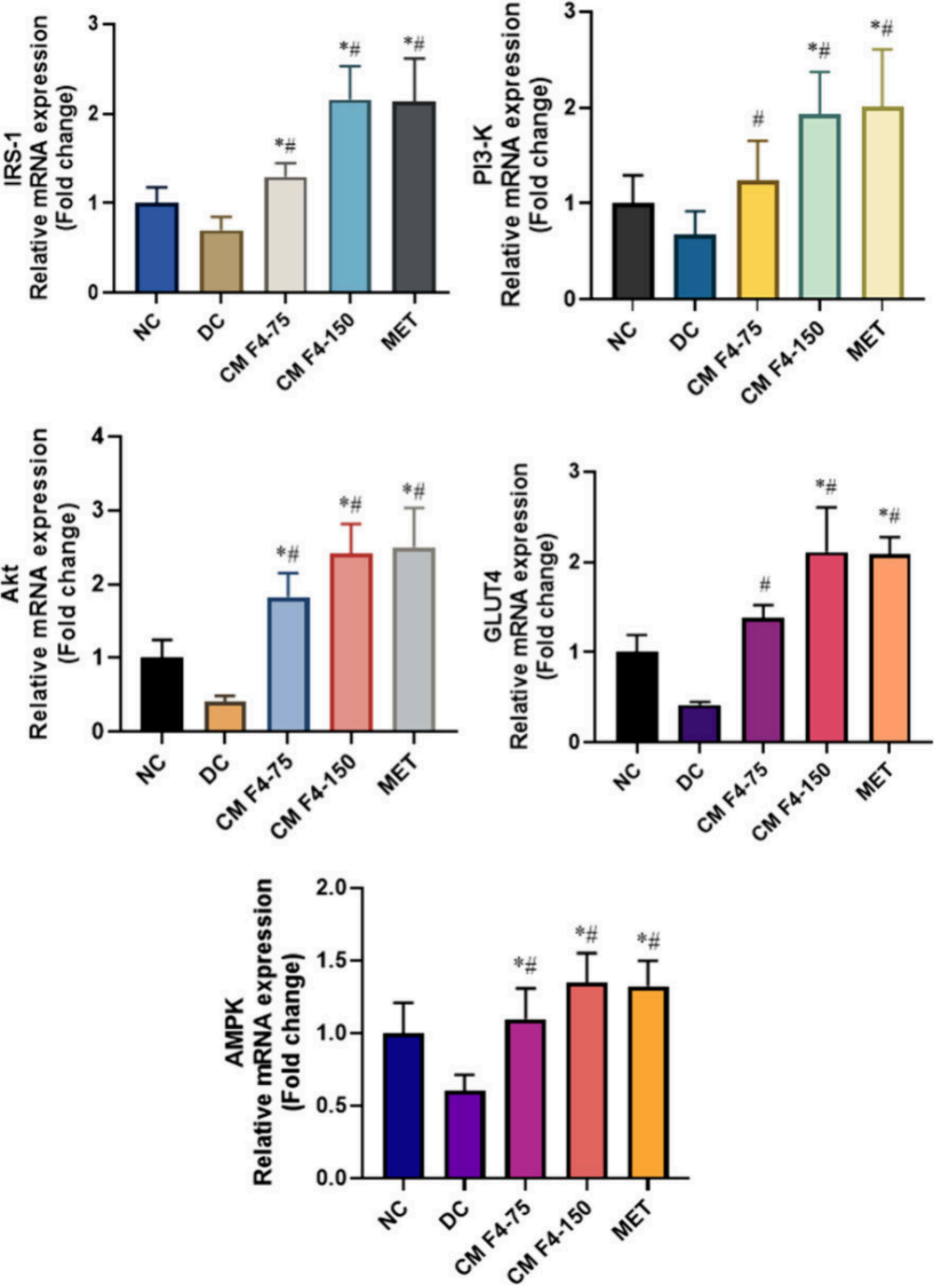
Effect of CM F4 on Molecular level gene expression in muscle tissue of HFD-STZ-induced T2DM rats. Relative mRNA levels are presented as fold changes compared to the Normal control (NC), which was assigned an arbitrary value of 1 in the graph. Each bar represents the mean of three independent experiments. GAPDH was used as the endogenous reference gene for normalization. *p < 0.05 is considered significant compared to the NC. ^#^ *p* < 0.05 is considered significant compared to the DC.

### 2.13 Pathway Elucidation of Improved Glucose Metabolism

To determine whether the effects of CM F4 on mRNA expression were reflected at the protein level, Western blot analysis was performed. For comparative analysis, the same set of genes involved in insulin signaling and insulin-independent signaling pathways were investigated for their protein expression levels. The results revealed that the phosphorylation levels of IRS-1 in tyrosine (pIRS-1), PI3K (pPI3K), and the expression of GLUT were significantly decreased in diabetic control (DC) rats, indicating impaired insulin signaling. Interestingly, treatment with CM F4, particularly at the dose of 150 mg/kg, showed an elevated level of phosphorylation in the above insulin signaling genes, thereby contributing to the reduction of hyperglycemia (Figure 13). Similarly, the DC group also exhibited downregulation of the insulin-independent protein pAMPK. Remarkably, this downregulation was substantially improved in the pretreated groups, especially in the CM F4–150 group. The activation of the energy-sensing AMPK protein could promote the recruitment of the AS160 regulator.^78^ This signaling cascade ultimately contributes to the stimulation of GLUT4 translocation to the cell membrane. A study reported that the administration of tinosporaside to db/db mice led to an improvement in glucose tolerance and peripheral insulin sensitivity. These beneficial effects were associated with increased gene expression and phosphorylation of the markers related to PI3K and AMPK signaling pathways in the skeletal muscle tissue.^75^ Several *in vivo* studies have reported that glucose homeostasis can be achieved through the activation of both the PI3K/Akt pathway^79,80^ and the AMPK pathway.^78,81^ The results of the current study clearly indicate the potential of the CM F4 formulation to improve glucose metabolism in HFD-STZ-induced type 2 diabetic rats. This improvement appears to be mediated through both the insulin-dependent (PI3K-Akt) and insulin-independent (AMPK) signaling pathways. The proposed mechanism of action of CM F4 based on the summarized results, as illustrated in Figure 14

**Figure 13:**
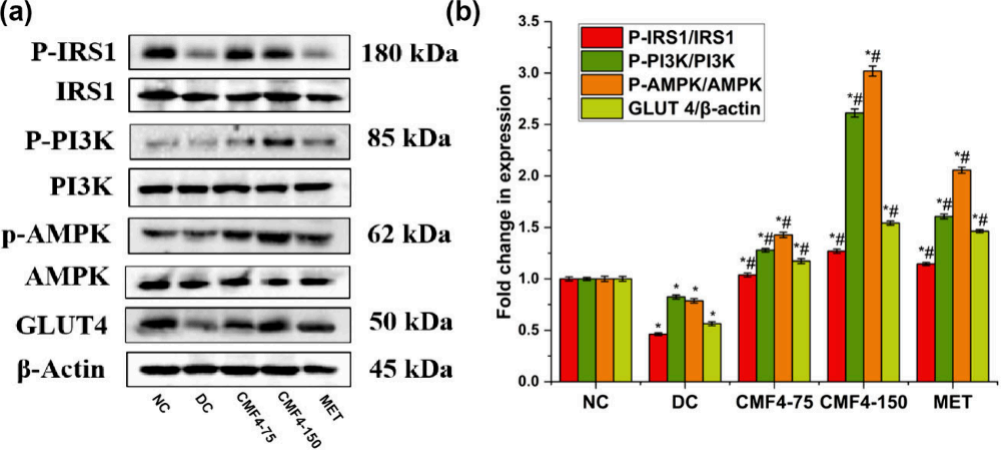
CM F4 administration in HFD-STZ-induced T2DM rats improved glucose metabolism via activating both the IRS and AMPK pathways. (a) Western blot analysis of IRS, PI3K, and AMPK, along with their phosphorylated forms and GLUT4 expression. (b) Fold change in expression compared with the NC and DC groups. β-actin was used as a control. Data are shown as mean ± SD (*n* = 3). *p < 0.05 is considered significant compared to the NC group. ^#^ *p* < 0.05 is considered significant compared to the DC group.

**Figure 14:**
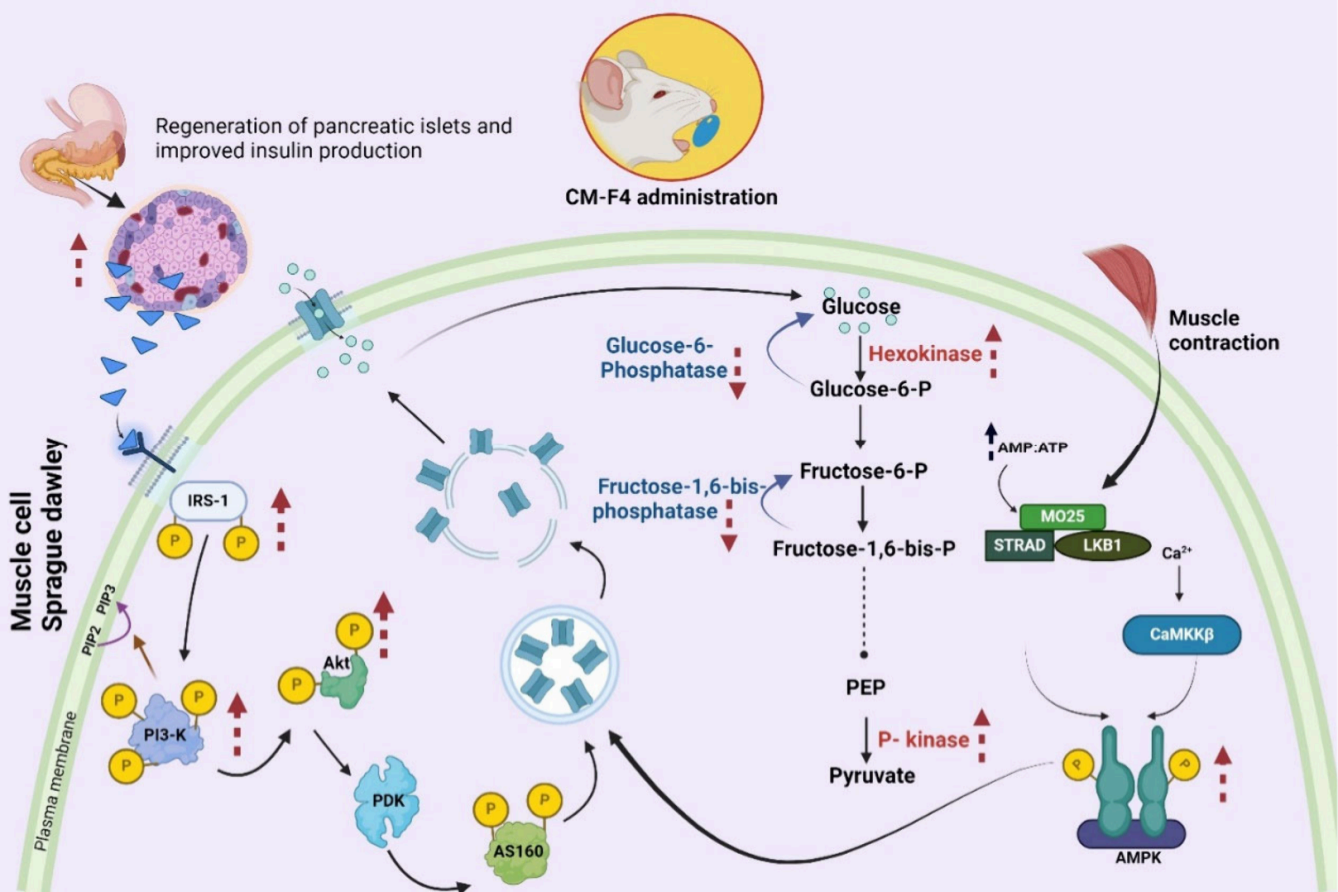
Proposed mechanism of action for CM F4 illustrates its antidiabetic effects with upregulation (↑) and downregulation (↓) of key biological targets. The potential of CM F4 involves a significant reduction in blood glucose levels and improved glucose tolerance, attributed to the regeneration or rejuvenation potential of pancreatic β-cells. These effects are quantitatively supported by mathematical indices, such as HOMA-IR, HOMA-β, and QUICKI, which confirm enhanced insulin sensitivity and restored β-cell function. CM F4 also modulates carbohydrate metabolism by upregulating glycolytic enzymes (hexokinase, pyruvate kinase) and downregulating gluconeogenic enzymes (glucose 6-phosphatase, fructose 1,6-bisphosphatase), contributing to better glucose homeostasis. At the molecular level, the formulation upregulates key genes and proteins in the PI3K/AKT and AMPK pathways, further promoting glucose uptake and insulin signaling. Created with BioRender, https://BioRender.com/6j6rfgd.

## 3 Conclusion

The CM F4 tablet formulation, developed from *Cassia mimosoides* ethyl acetate extract (CM-EA), represents a novel approach to enhancing the bioavailability of natural antidiabetic FCGs. The significant improvement in the dissolution profile achieved through the optimized wet granulation process marks a key advancement in addressing the pharmacokinetic challenges of herbal formulations. This enhanced dissolution directly translated to improve *in vivo* efficacy, as evidenced by the notable reduction in blood glucose levels, improved insulin sensitivity, and enhanced β-cell function in the T2DM rat model. Importantly, the modulation of both insulin-dependent (PI3K/Akt) and insulin-independent (AMPK) pathways underscores the multitargeted mechanism of action of CM F4, distinguishing it from conventional single-target therapies. The stability of key bioactive FCGs within the formulation also ensures the preservation of their therapeutic potential, contributing to the sustained efficacy observed in the preclinical model. This work not only validates the use of *Cassia mimosoides* in traditional medicine but also paves the way for future formulation innovations that can further enhance the therapeutic profile of natural products such as FCGs.

## 4 Experimental Section

### 4.1 Chemicals

The chemicals of analytical reagent grade were used in the entire study. Biochemical parameters were evaluated using kits purchased from Erba Lachema, Germany. Povidone K30, Crospovidone, Sodium Lauryl Sulfate, and Poloxomer were supplied by BASF Corporation in the USA. Starch and Sodium starch glycolate were provided by Roquette in France. Microcrystalline cellulose was supplied by FMC Corporation in the USA. DFE Pharma in India provided lactose, while Ferropharma in Germany supplied magnesium stearate. Imerys in France provided talc, and Innophos Holdings in the USA supplied dicalcium phosphate. Streptozotocin, protease inhibitor cocktail, was obtained from Sigma-Aldrich St. Louis, MO, USA. RIPA buffer was purchased from HiMedia Laboratories Private Limited, Maharashtra, India. Primary and secondary antibodies were purchased from Cell Signaling Technology (CST), Danvers, Massachusetts, USA.

### 4.2 Evaluation of Quality Control Parameters

The quality assessment of the plant material involved analyzing physicochemical parameters such as total ash, water-soluble ash, acid-insoluble ash, and extractive values (alcohol-soluble and water-soluble). The FCGs enriched ethyl acetate extract from CM was evaluated for its organoleptic characteristics like color, odor, texture, and taste. These tests were conducted following the guidelines set forth by the World Health Organization.^82^

### 4.3 Preparation of the FCGs Enriched CM-EA Extract

The plant was collected from the University of Kerala campus in Thiruvananthapuram and authenticated by the late Dr. M. Kamarudheen Kunju, Professor and Taxonomist, Department of Botany, Kariavattom. A voucher specimen has been deposited in the Kariavattom campus herbarium (ID: KUBH 10504). The aerial parts were cleaned and washed thoroughly with distilled water to remove impurities. The shade-dried material underwent hydro-alcoholic extraction in a solvent proportion 1:1. The mixture was allowed to stand for a specified duration before being filtered to obtain the crude extract. Subsequently, the ethanol–water extract was further fractionated using a liquid–liquid fractionation technique to obtain the ethyl acetate extract, which served as the API for the tablet formulation.

### 4.4 Drug-Excipient Compatibility Test by FTIR Analysis

To assess the compatibility of the extract with different pharmaceutical excipients, binary mixtures (1:1 w/w) were prepared. CM-EA and each excipient were individually ground using a mortar and pestle for approximately 5 min. A 1:1 ratio was selected to optimize the detection of potential interactions. Fourier-transform infrared (FTIR) spectroscopic analysis was performed on each excipient, CM-EA, as well as their respective blends. The aim was to obtain FTIR spectra over the range of 400 to 4000 cm^–1^. Spectral data were collected initially and again after one month, with the two spectra superimposed to identify any changes in the banding patterns over time.^83^

### 4.5 Formulation Development for Tablets

We implemented a modified wet granulation method to develop the tablet, following the procedure outlined by Narang.^83^ Four batches (F1–F4) of different excipient combinations were tried to make the tablets (Table 5). Briefly, measured quantities of the ingredients (Intragranular) and pulverized API were passed through a 40 mm sieve and mixed thoroughly. A binder solution of starch was added to the mix to form the granules and allowed to dry in an oven at 50 °C. The dried granules were passed through sieve number 20 to be made uniform. Finally, the granules and extragranular excipients were blended in a poly bag for 5 min. The tablets were compressed in an 8-station tablet compression machine (Cronimach, Gujarat, India) with a tablet punch having a dimension of 16 × 8 mm.

**Table 5:** Composition of Different Formulations of CM Tablets^a^

S. No.	Ingredients	Category	CM F1 Weight (mg)/tab	CM F2 Weight (mg) /tab	CM F3 Weight (mg) /tab	CM F4 Weight (mg) /tab
1	CM-EA	API	100	100	100	100
2	MCC	Diluent	170	170	170	175
3	Lactose	Diluent	167.5	-	195	-
4	DCP	Diluent	-	195	-	175
5	STH	Binder	10	10	10	5
6	PMR 407	Solubilizer	37.5	-	-	25
7	SLS	Solubilizer	-	10	10	-
8	SSG	Disintegrant	10	10	10	10
9	MSE	Lubricant	5	5	5	10
	Total (mg)		500	500	500	500

a CM-EA, *Cassia mimosoides*-Ethyl acetate extract; MCC, Microcrystalline cellulose 101; DCP, Dibasic calcium phosphate; STH, Starch; PMR 407, Poloxomer 407; SLS, Sodium Lauryl Sulfate; SSG, Sodium starch Glycolate; MSE, Magnesium stearate.

### 4.6 Evaluation of the Flow Properties

Before compression, the powder blends from all the test batches were analyzed, and their flow properties, including bulk density, tap density, compressibility index, Hausner’s ratio, and angle of repose, were evaluated.^84^

Bulk density (B.D): The bulk density of the powder was determined by following the procedure outlined below. The first step was to fill a graduated cylinder with a precise amount of powder. The volume of the powder in the cylinder was then measured, after which the weight of the powder was determined using a balance. The bulk density of the powder was calculated by using the formula:

Bulk density=Mass of powder/volume of the powder in the graduated cylinder

Tapped density (T.D): Tapped density of the granules was measured using a tap density tester. A known mass of powder was placed in a graduated cylinder attached to the apparatus. The apparatus was tapped from a height of 14 mm with an additional 3 mm on the surface for 100 taps or until a constant volume was reached. The tapped density was then calculated.

Tapped density(T.D)=Mass of
the powder/Tapped
volume

Hausner’s ratio and Carr’s index (Compressibility index): The indexes were used to evaluate the flow characteristics and cohesive nature. These parameters are derived from the measurements of the bulk density and the tapped density of the granules.

Carr’s index:(T.D−B.D/T.D)×100;⁣Hausner’s
ratio:T.D/B.D

The angle of repose was measured using the fixed height method. Granules were allowed to flow freely from a funnel held 2 cm above a horizontal surface, forming a cone. The diameter of the cone base was measured, the granules removed, and the average diameter calculated. The angle of repose (θ) was then determined using the cone’s height (h) and radius (r) with the formula: θ = tan^–1^(h/r).

### 4.7 Quality Assessment of the Tablets

All evaluations were performed using standardized instruments and procedures as per the guidelines outlined in the Indian Pharmacopoeia.^85^ Furthermore, additional research by Gupta and Moein^86,87^ supported the assessments.

Weight variation: To perform a weight variation study, 20 tablets from the batch were randomly selected, and the average weight of the selected tablets was calculated. Each tablet from the sample was weighed, and the weight of each tablet was compared with the average weight calculated in the previous step. Any variations in weight for each tablet were analyzed to determine the extent of weight variation within the batch.

Determination of hardness of tablets: The hardness of the tablet was measured using an Electrolab EDT (Electrolab India Pvt. Ltd.) apparatus. The tablet was placed between the plungers of the tester to ensure proper alignment. This is followed by the use of the threaded bolt to apply pressure and force the upper plunger against the spring until the tablet fractures. The reading displayed on the scale of the hardness tester was recorded when the tablet breaks.

Determination of friability of tablets: To determine the friability of tablets, a total of 20 tablets were initially weighed. Subsequently, these tablets were introduced into a plastic chamber of the Roche friabilator, where the apparatus was set in motion, revolving at a speed of 25 rotations per minute for 4 min. Following the completion of this period, the tablets were carefully removed from the chamber, carefully dusted, and subsequently reweighed. The discrepancy in weight between the initial and final measurements was then calculated.

### 4.8 *In Vitro* Dissolution and Disintegration Tests for the Formulated Tablets

Disintegration Test: A disintegrating apparatus (Type: ZT3/1, Erweka R GmbH, Heusenstamn, Germany) at a temperature of 37 ± 2 °C was used to carry out the disintegration test on tablets. The disintegration medium utilized in this study was distilled water. To prevent floating, a disk was positioned on each capsule. The duration required for all six tablets to disintegrate in the medium was recorded.^85^

*In vitro* dissolution studies (Drug Release): The study utilized the USP Dissolution Apparatus 2, manufactured by Veego in India, for experimenting and followed a modified protocol from Wang.^88^ A solution of 0.1 M HCL with a pH level of 1.2 served as the dissolution medium. The apparatus was set to operate at a speed of 100 rpm and maintained a temperature of 37 ± 0.5 °C. To simulate real-life conditions, three capsules from each formulation were sequentially added to separate round-bottom beakers at 5 min intervals. The experiment was conducted at different time intervals (15, 30, 60, and 120 min). At specified intervals, 10 mL aliquots were withdrawn and replaced with fresh dissolution medium maintained at 37 ± 0.5 °C. The withdrawn samples were filtered and analyzed via UV spectrophotometry at 296 nm. The cumulative drug release was calculated and plotted against time.

### 4.9 HPLC Quantification of Bioactive FCGs in Herbal Tablets

The stability and integrity of the four previously isolated bioactive FCGs such as orientin, isoorientin, diosmetin, and luteolin within the final tablet batch was assessed using a validated high-performance liquid chromatography method. To compare the content of the CM F4 tablet with the original CM-EA extract, a standard solution for each was prepared. One CM F4 tablet containing 100 mg of CM-EA extract was transferred to a volumetric flask and dissolved in 25 mL of methanol, resulting in a final concentration of 4 mg/mL of the CM-EA extract. A standard solution of the CM-EA extract was prepared at the same concentration (4 mg/mL) in methanol for comparative analysis. The HPLC system consisted of a Quaternary Solvent Manager-R, Sample Manager FTN-R, 2998 PDA Detector, and a Rheodyne injector equipped with a 50 μL volume loop. Chromatographic separation was achieved on a Waters Sunfire C18 reverse-phase column (250 mm × 4.6 mm, 5 μm particle size) maintained at 30 °C. A gradient elution program was employed using a mobile phase comprising solvent A (0.1% orthophosphoric acid in water) and solvent B (methanol) at a flow rate of 1 mL/min. The gradient program was as follows: 80% A for 20 min, followed by a linear decrease to 60% A over 10 min, 40% A over 10 min, 10% A over 5 min. The total run time was 45 min per sample. Detection was performed at 254 nm. Compound quantification was based on calibration curves generated using standard compounds at concentrations ranging from 250 to 1000 ppm. Peak identification was achieved by comparing retention times with those of authentic standards.

### 4.10 Experimental Animals

Sprague–Dawley male rats (170–200 g) were procured from Sree Chitra Tirunal Institute for Medical Sciences and Technology, Trivandrum, and work was carried out in the Department of Biochemistry, University of Kerala. The animals were acclimatized under standard environmental conditions (temperature 24–25 °C, 50 ± 10% relative humidity, and a 12-h/12-h light-dark cycle) for 1 week before experiments. All animals were fed standard rat chow and water ad libitum during acclimatization. All experimental protocols were reviewed and approved by the Institutional Animal Ethics Committee Kerala University [IAEC 18-KU-18/2022-BCH-SM (47)] for control and supervision of experiments on animals.

### 4.11 Induction of High-Fat Diet/Streptozotocin (HFD/STZ) Rat Model of T2DM

To induce a diabetic state, male rats were provided with modified HFD^89^ for 2 weeks (Supplementary File, Table S11). Following the feeding period, the rats were administered an intraperitoneal injection of a low dose (25 mg/kg body weight) of STZ that was dissolved in 0.1 M citrate buffer (pH 4.5) immediately before use.^90^ Prior to STZ administration, the animals had overnight access to 5% glucose in their drinking water to prevent drug-induced hypoglycemia. Subsequently, after a week of the injection, fasting blood glucose (FBG) levels were determined from tail blood samples using OneTouch Select Plus (LifeScan, California, USA). The induction and reduction of T2DM were confirmed by the Oral Glucose Tolerance Test (OGTT) and the blood glucose level (>250 mg/dL).

### 4.12 Experimental Design

To determine the safety profile, a dose-dependent toxicity study was conducted using the final tablet batch at different concentrations (5, 50, and 300 mg/kg body weight). Based on the evaluation of renal and toxicity markers and histopathology, two doses (75 mg/kg and 150 mg/kg body weight) were chosen for the mainstream study. The experimental animals were divided into five groups, comprising six rats each. The study design incorporated five distinct treatment groups. The normal control (NC) group received a standard pellet diet. The diabetic control (DC) group was provided an HFD and STZ to induce a diabetic state. Two HFD and STZ-induced diabetic groups were administered different dosages of the test formulation, designated as CM F4–75 and CM F4–150, at 75 mg/kg and 150 mg/kg of body weight, respectively. Finally, an HFD and STZ-induced diabetic group received the standard antidiabetic drug, metformin (MET), as the reference treatment. After the experimental period, the animals were sacrificed, and their serum and tissues were collected in containers and kept at −80 for further analysis. The tissue specimens of different organs were specifically collected and preserved in formalin for histopathological studies.

### 4.13 Estimation of Body Weight, Blood Glucose Level, and OGTT

Rat body weight and blood glucose levels were monitored throughout the 28-day study period at specific intervals (Days 0, 7, 14, 21, and 28). After a 12-h overnight fast, rats were orally administered a glucose solution (1 g/kg body weight) via gavage. Blood glucose measurements were taken with a glucometer at 0, 30, 60, and 120 min postadministration. The incremental area under the curve was then calculated using a standard method.^90^

### 4.14 Hepatic and Renal Markers

The quantification of insulin was carried out by employing a rat Enzyme-Linked Immunosorbent Assay (ELISA) kit provided by Merck, Germany. Markers indicative of hepatic toxicity, including Alanine Transaminase (ALT), Aspartate aminotransferase (AST), Alkaline Phosphatase (ALP), and Gamma Glutamyl Transferase (GGT), and renal toxicity, such as Urea, Uric acid, and Creatinine, were assessed. All the assays are done using commercially supplied kits from Erba Lachema, Germany.

### 4.15 Carbohydrate Metabolizing Enzymes

The activity levels of glycolytic enzymes (hexokinase (EC 2.7.1.1)^91^ and pyruvate kinase (EC 2.7.1.40)^92^) and hepatic gluconeogenic enzymes (glucose-6-phosphatase (EC 3.1.3.9)^93^ and fructose-1,6-bisphosphatase (EC 3.1.3.11)^94^) were measured.^68^

### 4.16 Insulin Resistance and β-Cell Function: Key Indicators

In this particular investigation, the assessment of insulin resistance, β cell function, and insulin sensitivity was conducted through three distinct methodologies: the homeostasis model HOMA-IR (Homeostasis Model Assessment for Insulin Resistance), HOMA-β (Homeostasis Model Assessment for β cell function), and the sensitivity check index, QUICKI (Quantitative Insulin Check Index).^47,53^ This particular approach entails the quantification of the fundamental levels of glucose and insulin, which are subsequently employed in mathematical equations to procure the outcomes for evaluating the functions.

- HOMA-IR = [fasting serum insulin (μU/mL) x fasting blood glucose (mmol/L)]/22.5
- HOMA-β = [(fasting serum insulin in μU/mL x 20)/(fasting blood glucose in mmol/L – 3.5)].
- QUICKI = 1/[log (fasting serum insulin in μU/mL) + log (fasting blood glucose in mmol/L).

### 4.17 Western Blot Analysis in Rat Skeletal Muscle Cells

To elucidate the molecular mechanisms underlying the effects of CM F4 on glucose metabolism, the expression of key insulin signaling markers in insulin-sensitive tissues were analyzed.^95^ Frozen skeletal muscle tissues were pulverized in liquid nitrogen and then homogenized in 1 mL of cold RIPA lysis buffer containing a protease inhibitor cocktail. The homogenate was subjected to intermittent vortexing for 30 min in an ice bath and then centrifuged at 12,000 g for 15 min at 4 °C. Total protein concentration was determined using a BCA protein assay kit. Lysates were separated via SDS-PAGE and transferred to PVDF membranes. The membranes were blocked with 5% skim milk for 2 h at low temperature before being incubated overnight with primary antibodies (1:1000 dilution). Following this, the membranes were incubated with HRP-conjugated secondary antibodies (1:10,000 dilution). Protein bands were visualized using the ECL substrate kit (Takara, Kusatsu, Japan).

### 4.18 RT-PCR Analysis

Total RNA was extracted and reverse transcriptase-polymerase chain reaction (RT-PCR) was performed according to a previously established protocol.^77^ Total RNA was isolated from skeletal muscle tissue (100 mg) of normal and treated diabetic rat groups using RNAiso Plus (Takara, Kusatsu, Japan). Purity and concentration were assessed using a Nanodrop spectrophotometer (Thermo Fisher Scientific Inc., Waltham, MA, USA). Complementary DNA (cDNA) was synthesized from 1 μg of total RNA using a Takara RT-PCR kit (Takara, Kusatsu, Japan). Real-time PCR amplification (CFX96 real-time PCR detection system) was performed using rat-specific primers (listed in Table 6) under the following conditions: 95 °C for 30 s, 55 °C for 30 s, and 72 °C for 60 s (40 cycles). GAPDH served as the reference gene. The ΔCt value represents the difference between target and housekeeping gene threshold cycles. Experiments were performed in triplicate, and results are expressed as fold changes in target gene expression relative to the reference, normalized to GAPDH.

ΔΔCt=ΔCtof untreated sample−ΔCtof sample

**Table 6:** List of Primer Sequences used for RT-PCR

Genes	Forward primer	Reverse primer
IRS-1	5′-GGATGCAAGTGGATGACTC −3′	5′-CGGAGGATTGTTGAGATGGT-3′
PI3K	5′-TTAAACGCGAAGGCAACGA-3′	5′-CAGTCTCCTCCTGCTGTCGAT-3′
Akt	5′-CCGCTATTATGCCATGAAGAT-3′	5′-TGTGGGCGACTTCATCCT-3′
GLUT4	5′ACAATGTCTTGGCTGTGCTG-3′	5′-TCCCACATACATAGGCACCA-3′
GAPDH	5′CAACTCCCTCAAGATTGTCAGCAA-3′	5′-GGCATGGACTGTGGTCATGA-3′

### 4.19 Histopathological Analysis of Pancreas

The pancreatic specimens were immersed in a 10% neutral formalin solution for 48 h to facilitate preservation. Subsequently, the fixative solution underwent dehydration using various ethyl alcohol and water mixtures, followed by cleaning with xylene. Following paraffin embedding, 5 μm-thick pancreatic sections were obtained using a rotary microtome. These sections were stained with hematoxylin and eosin and mounted in a xylene-based mounting medium for microscopic examination.^96^

### 4.20 Statistical Analysis

Statistical analysis was performed using the SPSS statistical analysis program (version 17) using a one-way variance analysis. Significant differences among groups were determined using Tukey’s range test. A probability (*p*) value of less than 0.05 was considered indicative of statistical significance.

## Supplementary Material



## Notes

Details of flow property evaluation of the formulated tablets; body weight changes during the treatment period; blood glucose levels in experimental groups; oral glucose tolerance test (OGTT) data with time-point measurements; and mRNA expression analysis of genes involved in glucose metabolism and insulin signaling (PDF)
