# Supplementary material for: A Novel Flavonoid C‑Glycosides Integrated Tablet for Improved Dissolution, Pancreatic Repair, and Insulin Mediated Glucose Regulation in Type 2 Diabetic Rats
Source: ACS Omega. 2025 Sep 17;10(38):44510–27. doi: 10.1021/acsomega.5c06592 (PMC12489731; doi:10.1021/acsomega.5c06592)
Supplement: Supplementary file 1 [file ao5c06592_si_001.pdf]

A novel flavonoid C-glycosides integrated tablet for improved dissolution, pancreatic repair, and insulin mediated glucose regulation in type 2 diabetic rats

Abdul Rahim Muhammed Jasim [1,2], Anithakumari Aswathy Krishna [1,2], Beena Levakumar Abhirami [1,2], Alaganandam Kumaran[1,2*], Chun-Hui Chiu[3,4*]

[1]Agro and Food Processing Technology Division, CSIR–National Institute for Interdisciplinary Science and Technology (NIIST), Thiruvananthapuram 695019, Kerala, India

[2]Academy of Scientific and Innovative Research (AcSIR), Ghaziabad 201002, India.

[3]Research Center for Food and Cosmetic Safety, College of Human Ecology, Chang Gung University of Science and Technology, Taoyuan City, 333324, Taiwan.

[4]Department of Nephrology, Chang Gung Memorial Hospital, Taoyuan City, 333008, Taiwan.

*Corresponding author

Alaganandam Kumaran, Agro and Food Processing Technology Division, CSIR National Institute for Interdisciplinary Science and Technology, Thiruvananthapuram, 695019, Kerala, India. Email: akumaran@niist.res.in,

Chun-Hui Chiu Address: No. 261, Wenhua 1st Road, Kwei-Shan District, Taoyuan City 333324, Taiwan, R.O.C, E-mail: chchiu@mail.cgust.edu.tw, Tel: 886-3-211-8999; Fax: 886-3-211-8866

Supplementary data

Table of Contents

Table S1. Flow properties (R1-R3, Triplicate value of experiment)……………………**2**

Table S2. HPLC method validation parameters (Calibration data-based)………………**3**

Table S3. Estimation of body weight…………………………………………………**3**

Table S4. Estimation blood glucose level ……………………………………………**4**

Table S5. OGTT (Blood glucose level, 0-120 min)……..………………………………**5**

Table S6-S10. mRNA expression level ……..…………………………..……………**6**

Table S11. . Composition and caloric contribution of the High-Fat Diet (HFD) ………**7**

Table S1. Flow properties (R1-R3, Triplicate value of experiment)

Bulk density (g/ml)					
	R1	R2	R3	Mean	SD
CM F 1	0.50	0.54	0.51	0.52	0.024
CM F 2	0.68	0.79	0.69	0.72	0.06
CM F 3	0.68	0.75	0.69	0.71	0.03
CM F 4	0.69	0.74	0.70	0.71	0.02
**Tapped density (g/ml)**					
	R1	R2	R3	Mean	SD
CM F 1	0.66	0.74	0.67	0.693266667	0.04
CM F 2	0.87	0.99	0.89	0.92	0.06
CM F 3	0.79	0.91	0.78	0.83	0.07
CM F 4	0.79	0.87	0.79	0.82	0.04
**Compressibility index (%)**					
	R1	R2	R3	Mean	SD
CM F 1	23.12	24.43	22.99	23.52	0.79
CM F 2	21.76	22.34	21.70	21.94	0.35
CM F 3	14.00	14.92	13.91	14.28	0.55
CM F 4	13.54	14.36	13.46	13.79	0.49
**Hausner's ratio**					
	R1	R2	R3	Mean	SD
CM F 1	1.25	1.40	1.23	1.3	0.09
CM F 2	1.39	1.22	1.21	1.28	0.10
CM F 3	1.11	1.09	1.26	1.16	0.09
CM F 4	1.14	1.14	1.18	1.16	0.02
**Angle of Repose (°)**					
	R1	R2	R3	Mean	SD
CM F 1	35.53	38.04	35.28	36.29	1.52
CM F 2	36.09	32.66	32.31	33.69	2.08
CM F 3	34.32	35.16	34.24	34.58	0.51
CM F 4	31.50	33.27	31.63	32.14	0.98

Bioactive FCGs	Concentration (ppm)	Peak area	R^2	LOD (µg/mL)	LOQ (µg/mL)
Orientin	125	4807690	0.9999	2.73	8.28
	250	9368029			
	500	18528313			
	1000	36971475			
Isoorientin	125	3829883	0.9998	18.98	57.51
	250	6950907			
	500	13761129			
	1000	26419106			
Luteolin	62.5	3637858	0.9998	7.90	23.94
	125	7432060			
	250	15393310			
	500	31787580			
Diosmetin	62.5	3206120	0.9989	20.58	62.37
	125	6359217			
	250	12219931			
	500	22885611			

Table S2. HPLC method validation parameters (Calibration data-based)

The above data support the validation of the HPLC method used for quantification of flavonoid C-glycosides (Orientin, Isoorientin, Luteolin, and Diosmetin) in CM extract and tablets, following ICH Q2(R1) guidelines. Validation parameters include linearity, limit of detection (LOD), and limit of quantification (LOQ).

Table S3. Estimation of body weight

**NC**	**Day 0**	**Day 7**	**Day 14**	**Day 21**	**Day 28**
	83.76	91.65	93.86	90.60	87.50
	93.99	101.25	105.66	100.19	98.34
	90.27	97.76	101.37	96.71	94.40
	95.84	102.99	107.80	101.94	100.32
	85.62	93.40	96.01	92.34	89.47
	99.56	106.48	112.09	105.43	104.27
**Average**	91.51	98.92	102.80	97.87	95.72
**SD**	6.10	5.72	7.03	5.72	6.47
**DC**	**Day 0**	**Day 7**	**Day 14**	**Day 21**	**Day 28**
	306.37	345.67	387.29	410.68	426.01
	321.92	362.78	409.11	438.77	459.02
	316.26	356.56	401.18	428.56	447.01
	324.74	365.89	413.08	443.88	465.02
	309.19	348.78	391.26	415.79	432.01
	330.40	372.12	421.01	454.09	477.02
**Average**	318.15	358.63	403.82	431.96	451.01
**SD**	9.28	10.21	13.02	16.76	17.98

CM F4-75	Day 0	Day 7	Day 14	Day 21	Day 28
	457.00	395.58	303.14	193.89	170.43
	485.94	421.07	322.35	216.25	186.51
	475.42	411.80	315.37	208.12	180.66
	491.20	425.70	325.85	220.31	189.43
	462.26	400.22	306.63	197.96	173.35
	501.72	434.97	332.84	228.44	195.28
**Average**	478.92	414.89	317.70	210.83	182.61
**SD**	15.76	13.88	10.47	12.17	8.76
**CM F4-150**	**Day 0**	**Day 7**	**Day 14**	**Day 21**	**Day 28**
	470.78	377.97	295.68	219.36	106.48
	497.46	401.51	318.28	241.02	123.85
	487.76	392.95	310.06	233.14	117.54
	502.31	405.79	322.39	244.96	127.01
	475.63	382.25	299.79	223.30	109.64
	512.01	414.36	330.61	252.84	133.33
**Average**	490.99	395.81	312.80	235.77	119.64
**SD**	14.53	12.82	12.31	11.80	9.46
**MET**	**Day 0**	**Day 7**	**Day 14**	**Day 21**	**Day 28**
	466.35	382.67	331.98	261.94	117.38
	496.26	407.88	354.05	282.76	133.50
	485.39	398.71	346.02	275.19	127.64
	501.70	412.46	358.06	286.54	136.43
	471.79	387.25	335.99	265.72	120.31
	512.57	421.62	366.09	294.11	142.29
**Average**	489.01	401.76	348.70	277.71	129.59
**SD**	16.29	13.73	12.02	11.34	8.78

Table S4 Estimation blood glucose level

NC	Day 0	Day 7	Day 14	Day 21	Day 28
	244.71	277.15	298.92	320.24	339.70
	258.86	288.80	307.79	330.43	348.65
	253.71	284.56	304.56	326.72	345.40
	261.43	290.91	309.40	332.28	350.28
	247.28	279.27	300.53	322.09	341.33
	266.58	295.15	312.62	335.98	353.53
**Average**	255.43	285.97	305.64	327.96	346.48
**SD**	8.44	6.95	5.29	6.08	5.34
**DC**	**Day 0**	**Day 7**	**Day 14**	**Day 21**	**Day 28**
	373.23	341.37	251.22	217.30	193.33
	385.76	351.20	259.44	225.87	199.48
	381.20	347.63	256.45	222.75	197.24
	388.04	352.99	260.94	227.43	200.59
	375.50	343.15	252.72	218.86	194.45
	392.59	356.57	263.93	230.54	202.83
**Average**	382.72	348.82	257.45	223.79	197.99

SD	7.48	5.87	4.90	5.11	3.67
**CM F4-75**	**Day 0**	**Day 7**	**Day 14**	**Day 21**	**Day 28**
	357.80	324.86	286.87	272.43	263.98
	369.34	338.31	296.55	278.33	277.96
	365.14	333.42	293.03	276.18	272.88
	371.43	340.75	298.31	279.40	280.51
	359.90	327.30	288.63	273.50	266.52
	375.63	345.64	301.83	281.55	285.59
**Average**	366.54	335.05	294.20	276.90	274.57
**SD**	6.88	8.02	5.77	3.52	8.34
**CM F4-150**	**Day 0**	**Day 7**	**Day 14**	**Day 21**	**Day 28**
	365.86	335.92	281.84	254.51	238.19
	374.45	346.95	291.91	263.98	245.83
	371.33	342.94	288.25	260.54	243.06
	376.02	348.95	293.74	265.71	247.22
	367.42	337.93	283.67	256.23	239.58
	379.14	352.96	297.40	269.15	250.00
**Average**	372.37	344.27	289.47	261.69	243.98
**SD**	5.13	6.58	6.00	5.65	4.56
**MET**	**Day 0**	**Day 7**	**Day 14**	**Day 21**	**Day 28**
	347.98	337.36	290.03	280.78	260.02
	356.99	343.46	301.58	290.14	267.53
	353.71	341.25	297.38	286.74	264.80
	358.62	344.57	303.68	291.85	268.90
	349.62	338.47	292.13	282.48	261.38
	361.90	346.79	307.87	295.25	271.63
**Average**	354.80	341.98	298.78	287.87	265.71
**SD**	5.37	3.64	6.89	5.59	4.48

Table S5. OGTT (Blood glucose level, 0-120 min)

NC	0 min	30 min	60 min	90 min	120 min
	106.12	155.23	137.81	118.75	108.39
	115.79	166.08	149.24	132.22	120.40
	112.27	162.14	145.09	127.32	116.03
	117.55	168.05	151.32	134.66	122.58
	107.88	157.21	139.89	121.20	110.57
	121.07	171.99	155.47	139.56	126.95
**Average**	113.45	163.45	146.47	128.95	117.49
**SD**	5.77	6.47	6.82	8.03	7.16
**DC**	**0 min**	**30 min**	**60 min**	**90 min**	**120 min**
	440.35	473.17	488.43	479.59	481.78
	459.36	491.01	508.60	502.38	508.65
	452.45	484.52	501.27	494.09	498.88
	462.82	494.25	512.27	506.53	513.54
	443.81	476.41	492.10	483.73	486.67
	469.73	500.74	519.61	514.81	523.31

**Average**	454.75	486.68	503.71	496.85	502.14
**SD**	11.34	10.64	12.03	13.60	16.03
**CM F4-75**	**0 min**	**30 min**	**60 min**	**90 min**	**120 min**
	174.50	224.27	211.71	176.11	155.52
	185.22	237.69	218.42	190.59	165.97
	181.32	232.81	215.98	185.32	162.17
	187.18	240.13	219.64	193.23	167.88
	176.45	226.71	212.93	178.74	157.42
	191.08	245.01	222.08	198.50	171.68
**Average**	182.62	234.44	216.80	187.08	163.44
**SD**	6.40	8.01	4.00	8.64	6.24
**CM F4-150**	**0 min**	**30 min**	**60 min**	**90 min**	**120 min**
	111.42	218.91	182.30	155.90	134.63
	122.30	234.42	196.65	167.88	146.35
	118.34	228.78	191.43	163.52	142.09
	124.28	237.24	199.25	170.06	148.48
	113.39	221.73	184.91	158.08	136.76
	128.24	242.88	204.47	174.41	152.75
**Average**	119.66	230.66	193.17	164.97	143.51
**SD**	6.49	9.25	8.56	7.15	6.99
**MET**	**0 min**	**30 min**	**60 min**	**90 min**	**120 min**
	122.62	229.69	201.72	157.54	133.11
	131.54	241.19	216.02	170.98	144.19
	128.30	237.01	210.82	166.10	140.16
	133.16	243.28	218.62	173.43	146.20
	124.24	231.78	204.32	159.99	135.12
	136.41	247.46	223.83	178.31	150.23
**Average**	129.38	238.40	212.56	167.72	141.50
**SD**	5.32	6.86	8.53	8.02	6.61

Table S6. mRNA expression level

**IRS-1**							
**Sample name**	**ΔΔCT**			**2(-ΔΔCt)**			**Mean $2^{(-\Delta\Delta Ct)}$**
Control	0	0	0	0	0	0	1 ± 0.17
DC	0.20	0.73	0.67	0.87	0.60	0.627	0.70 ± 0.14
CM F4-75	-0.22	-0.55	-0.33	1.16	1.46	1.25	1.29 ± 0.15
CM F4-150	-0.82	-1.32	-1.14	1.76	2.49	2.20	2.15 ± 0.36
MET	-1.42	-0.97	-0.82	2.67	1.95	1.76	2.13 ± 0.47

Table S7.

**PI3K**							
**Sample name**	**ΔΔCT**			**2(-ΔΔCt)**			**Mean $2^{(-\Delta\Delta Ct)}$**
Control	0	0	0	0	0	0	1 ± 0.29
DC	0.52	1.24	0.13	0.69	0.42	0.52	0.67 ± 0.24

CM F4-75	-0.56	0.37	-0.55	1.48	0.77	-0.56	1.24 ± 0.57
CM F4-150	-0.76	-1.28	-0.72	1.7	2.43	-0.76	1.93 ± 0.44
MET	-0.88	-1.41	-0.59	1.84	2.66	-0.88	2.01 ± 0.59

Table S8.

**Akt**							
**Sample name**	**ΔΔCT**			**2(-ΔΔCt)**			**Mean $2^{(-\Delta\Delta Ct)}$**
Control	0	0	0	0	0	0	1 ± 0.24
DC	1.16	1.61	1.1	0.44	0.32	0.46	0.41 ± 0.07
CM F4-75	-1.1	-0.58	-0.88	2.14	1.49	1.84	1.82 ± 0.32
CM F4-150	-1.07	-1.52	-1.19	2.09	2.86	2.28	2.41 ± 0.40
MET	-0.98	-1.33	-1.6	1.97	2.51	3.03	2.50 ± 0.52

Table S9.

**GLUT4**							
**Sample name**	**ΔΔCT**			**2(-ΔΔCt)**			**Mean $2^{(-\Delta\Delta Ct)}$**
Control	0	0	0	0	0	0	1 ± 0.19
DC	1.25	1.15	1.37	0.42	0.45	0.38	0.41 ± 0.03
CM F4-75	-0.54	-0.29	-0.56	1.45	1.22	1.47	1.38 ± 0.13
CM F4-150	-0.91	-1.42	-0.83	1.87	2.67	1.77	2.11 ± 0.49
MET	-1.14	-0.91	-1.13	2.2	1.87	2.18	2.09 ± 0.18

Table S10.

**AMPK**							
**Sample name**	**ΔΔCT**			**2(-ΔΔCt)**			**Mean $2^{(-\Delta\Delta Ct)}$**
Control	0	0	0	0	0	0	1 ± 0.20
DC	0.47	1.00	0.77	0.71	0.5	0.58	0.60 ± 0.10
CM F4-75	0.23	-0.68	0.28	0.85	1.6	0.82	1.09 ± 0.44
CM F4-150	-0.43	0.12	-0.82	1.35	0.92	1.76	1.34 ± 0.42
MET	-0.57	-0.42	-0.19	1.48	1.34	1.14	1.32 ± 0.17

Table S11. Composition and caloric contribution of the High-Fat Diet (HFD)

**Ingredient**	**Amount (g/kg diet)**	**Contribution**	**Caloric Contribution (kcal)**
Normal pellet diet (NPD)	500 g	Base (carbohydrate/protein)	1580 (1140 from carbs + 350 from protein + 90 from fat)
Lard	186 g	Fat source	1674
Dalda (hydrogenated fat)	43 g	Fat source	387
Cholesterol	10 g	Lipid modifier	0
Vitamin powder	115 g	Micronutrients	0
Casein powder	146 g	Protein source	584
Total (per 1 kg diet)	1000 g	–	4225
